# Supplementary material for: Epidemiology and risk factors of coronary artery aneurysm in Taiwan: a population based case control study
Source: BMJ Open. 2017 Jun 30;7(6):e014424. doi: 10.1136/bmjopen-2016-014424 (PMC5734584; doi:10.1136/bmjopen-2016-014424)
Supplement: Supplementary material 1 [file bmjopen-2016-014424supp001.pdf]

Supplementary

eTable 1. Spearman correlation coefficients among risk factors

		DM	CVD	PVD	VV	AD	AA	SLE	RA	IBD
**CAS**	r	0.103*	0.036*	0.023*	0.002	0.030*	0.021	0.010	0.015	-0.011
	p	**<0.0001**	**<0.05**	**<0.05**	0.873	**<0.05**	0.068	0.397	0.176	0.342
**HTN**	r	0.284*	0.198*	0.028*	-0.008	0.035*	0.040*	0.016	0.019	0.007
	p	**<0.0001**	**<0.0001**	**<0.05**	0.483	**<0.05**	**<0.001**	0.149	0.092	0.549
**Lipid**	r	0.267*	0.015	0.036*	-0.012	-0.007	0.010	0.002	0.007	-0.025*
	p	**<0.0001**	0.178	**<0.05**	0.301	0.515	0.375	0.831	0.532	**<0.05**

r, Spearman correlation coefficient; * $p < 0.05$;

CAS, coronary atherosclerosis; HTN, hypertension; Lipid, Dyslipidemia; DM, diabetes mellitus; CVD, cerebrovascular disease; PVD, peripheral vascular disease; VV, varicose vein; AD, aortic dissection; AA, aortic aneurysm; SLE, systemic lupus erythematosus; RA, rheumatoid arthritis; IBD, inflammatory bowel disease.
