# Supplementary material for: Epidemiology and risk factors of coronary artery aneurysm in Taiwan: a population based case control study
Source: BMJ Open. 2017 Jun 30;7(6):e014424. doi: 10.1136/bmjopen-2016-014424 (PMC5734584; doi:10.1136/bmjopen-2016-014424)
Supplement: Author's manuscript [file bmjopen-2016-014424.draft_revisions.pdf]

BMJ Open

# Epidemiology and risk factors of coronary artery aneurysm in Taiwan

| | |
|---|---|
| Journal: | *BMJ Open* |
| Manuscript ID | bmjopen-2016-014424 |
| Article Type: | Research |
| Date Submitted by the Author: | 23-Sep-2016 |
| Complete List of Authors: | Fang, Chein-Tang ; Kaohsiung Medical University<br>Fang , Yi-Ping ; Kaohsiung Medical University<br>Huang, Yaw-Bin; Kaohsiung Medical University, School of Pharmacy<br>CHEN, CHUNG-YU |
| <b>Primary Subject Heading</b>: | Epidemiology |
| Secondary Subject Heading: | Cardiovascular medicine |
| Keywords: | Coronary artery aneurysm, Epidemiology < TROPICAL MEDICINE, risk factor |
| | |

SCHOLARONE™
Manuscripts

For peer review only

For peer review only - http://bmjopen.bmj.com/site/about/guidelines.xhtml

# Epidemiology and risk factors of coronary artery aneurysm in Taiwan

Chein-Tang Fang [1], Yi-Ping Fang [1], Yaw-Bin Huang[1, 2],Chung-Yu Chen[1, 2]

1. School of Pharmacy, Master Program in Clinical Pharmacy, Kaohsiung Medical University, Kaohsiung, Taiwan.
2. Department of Pharmacy, Kaohsiung Medical University Hospital, Kaohsiung, Taiwan.

*Corresponding author: Chung-Yu Chen, School of Pharmacy,
No. 100, Shihcyuan 1st Rd., Sanmin District, Kaohsiung City 80708, Taiwan (R.O.C.)
Tel: 886-7-3121101 ext 2375
Fax: 886-7-3210683
E-mail: jk2975525@kmu.edu.tw
*Corresponding author: Yaw-Bin Huang, School of Pharmacy,
No. 100, Shihcyuan 1st Rd., Sanmin District, Kaohsiung City 80708, Taiwan (R.O.C.)
Tel: 886-7-3121101 ext 2166
Fax: 886-7-3210683
E-mail: yabihu@kmu.edu.tw

**Word count:** 3400

**Key words**: Coronary artery aneurysm**;** epidemiology; risk factor

For peer review only

## Abstract

**Objectives:** Coronary artery aneurysm (CAA) was a usually asymptomatic and rare diseases. There is limited epidemiological data of CAA in Asian or world.

**Design:** A retrospective case-cohort study

**Setting:** A population-based database study from the Taiwan's National Health Insurance Research Database, included participants between 2005 and 2013.

**Participants:** CAA patients by using diagnostic code (ICD: 414.11) with CAA examinations.

**Outcome measures:** The incidence and mortality of CAA were calculated. Furthermore, we also conducted matching non-CAA patients according to age, gender, index year at a 1:10 ratio to explore risk factors for CAA by logistic regression.

**Result:** A total of 1397 CAA patients were identified during 2005 to 2011. The average annual incidence and mortality of CAA in Taiwan were 0.87 and 0.05 per 100,000 population. Otherwise, the adjusted odds ratios (aOR) for coronary atherosclerosis, hypertension, dyslipidemia and diabetes were 7.97, 2.09, 2.48 and 1.51, respectively. Of note, aortic dissection (aOR: 6.76), aortic aneurysm (aOR: 5.82) and systemic lupus erythematosus (aOR: 4.09) were found significantly associated with CAA.

**Conclusion:** Epidemiology of CAA in Taiwan was low. Aside from cardiovascular risk factors, aortic diseases and systemic lupus erythematosus need to be further investigated in CAA patients.


For peer review only

## Strengths and limitations of this study

1. In this nationwide epidemiology study involving retrospective analysis of a claims database, we evaluated the epidemiology and risk factors for CAA in Asian country.
2. The major limitation of the study was no laboratory data on coronary angiography data and lifestyle habits of the patients.
3. As a retrospective observational analysis, this study only provides associative information from case-control study.

For peer review only

## Introduction

Coronary artery aneurysm (CAA) is a rare finding in patients referred for coronary angiography. It is characterized by abnormal dilatation of the vessel lumen defined as a lumen with diameter that exceeds more than 1.5-fold the diameter of the normal vessel lumens adjacent to it or as the largest coronary vessel lumen.[1] Previous case series on angiography have reported a wide range of prevalence from 0.2% to 6.0.[1-4] Two other case series in Taiwan indicated that 0.25%–2.6% of patients had CAA.[5, 6] Although CAA commonly coexists with atherosclerosis,[1, 7] various potential causes such as congenital, inflammatory and connective tissue disorders, and other factors have been noted in the literature.[8, 9] Among these risk factors, Kawasaki disease is well recognized as the main cause in children; it occurs predominantly in Asian countries.[10, 11] The pathogenesis of CAA is postulated to be the degradation of the extracellular matrix of the media by matrix metalloproteinases and is considered to be similar to that of other aneurysms in larger vessels.[12, 13] Instead of presenting as a benign entity, CAA shows the clinical manifestations of coronary artery diseases such as angina and acute coronary syndrome.[14-16] At present, information regarding CAA and its related risk factors is lacking in Asian countries. Accordingly, our aim was to investigate the epidemiology and risk factors for CAA in a Taiwanese population.

For peer review only

## Methods

### Data sources

We obtained specific dataset of all CAA patients in Taiwan from the NHIRD. All medical records comprising outpatient care, inpatient care, emergency care, and prescriptions from 2004 to 2012 were used for analysis in the epidemiologic study.

For the case-control study, we selected controls from the Longitudinal Health Insurance Database (LHID2005), which contained one million samples that were randomly selected from the NHIRD and were representative of 23 million people in Taiwan. This study was approved by the Institutional Review Board of Kaohsiung Medical University Hospital (KMUH-IRB-EXEMPT-20130199). Current NHIRD and hospital regulations and guidelines did not mandate informed consent in this retrospective case-control study. All procedures performed were in accordance with the ethical standards of the institutional research committee and the directives of the Declaration of Helsinki.

For peer review only

### Study population

To identify patients diagnosed with CAA, we used the International Classification of Diseases, Ninth Revision, Clinical Modification (ICD-9-CM) code 414.11. Patients who had two outpatient diagnoses or one inpatient diagnosis of CAA between January 1, 2005 and December 31, 2011were identified. The index date was defined as the date of first diagnosis of

CAA. Patients who were diagnosed with CAA based on imaging studies, including coronary angiography, echocardiography, computed tomography, magnetic resonance imaging, and cardiac catheterization, within 90 days (3 months) before or after the index date were enrolled.

In the case-control study, cases included adult subjects who were hospitalized with a diagnosis of CAA between January 1, 2005 and December 31, 2011. Patients younger than 20 years and those who were diagnosed with CAA in the outpatient clinic were excluded. In the case group, the first hospitalization date for CAA was assigned as the index date. For the control selection, 10 patients were randomly selected for each case by matching age, sex, and index year of case diagnosis. The first admission date within the same year of CAA diagnosis was assigned as the index date for the control group.

For peer review only

**Survival status**

According to the validation by Cheng et al. of in-hospital mortality in the NHIRD,[17] we defined death as “4” or “A” status record upon discharge and “disenrollment” insurance status in the Registry for Beneficiaries. On the other hand, patients admitted because of an emergent reason, but without follow-up data were considered as death, which was also confirmed by checking the insurance status.

**Covariates**

In the epidemiologic study, we analyzed the age and sex, characteristics of subjects with

respect to geographical region, urbanization, and monthly insurance premiums in New Taiwan Dollars. In the case–control study, covariates for analysis included demographics and related risk factors.[8, 18-23] A period of one year before the index date was reserved for covariate evaluation in both groups.

**Statistical analysis**

Incidence, which was presented as number per 100,000, was defined as the number of patients with a newly-diagnosed CAA divided by the total Taiwanese population in each year. Mortality, which was presented as number per 100,000, was defined as the number of patients who died divided by the total Taiwanese population in each year. For trend analysis, the Cochran–Armitage test was used to detect any significant change in incidence or mortality during the study period. The demographic data of total population in Taiwan were obtained from the Statistics, Department of Household Registration, Ministry of the Interior[24].For descriptive statistics, continuous variables were presented as mean ± SD; categorical variables were presented as number and proportion (%).

The odds ratio and 95% confidence interval (95% CI) for the CAA were estimated by conditional logistic regression analysis for matched pairs data and were adjusted for the study covariates. The results were expressed as three models adjusted for different variables: Model 1, demographics and traditional cardiovascular diseases, except diabetes; Model 2,

For peer review only

demographics, diabetes and other risk factors; and Model 3, all variables mentioned above. Spearman's correlation coefficient was used to detect collinearity among variables. Data processing and statistical analysis were performed with SAS version 9.4 (SAS Inc., Cary, NC).

**Results**

**Epidemiologic study population**

From 2005 to 2011, there were 1493 patients who had at least two CAA diagnoses at an outpatient setting or one CAA diagnosis at an inpatient setting. After excluding 96 subjects who did not have any record of related examinations, a total of 1397 patients with CAA diagnosis were enrolled in our study (**Figure 1**). Among the 1397 patients, mean age was 37.76 ± 31.45years. There were 586 pediatric patients (age <20 years), 430 adults (age≥20 to<65 years), and 381 elderly patients (age ≥65 years), accounting for 41.9%, 30.8%, and 27.3% of the CAA population, respectively (**Table 1**). **Figure 2** shows the detailed age distribution, where in majority of pediatric patients were<5 years of age; in particular, patients who were ≤1 year old comprised 229 patients (23.2%). Furthermore, a male predilection (68.5%) was found, with a male to female ratio of 2.18. Most of the patients with CAA lived in northern Taiwan (39.1%), followed by central (37.1%), southern (22.0%), and

For peer review only

Table 1. Baseline characteristics of CAA population

| Variable (N=1397) | Number (%) |
|---|---|
| **Age, year (mean ± SD)** | 37.76 ± 31.45 |
| **Age group (year)** | |
| < 20 | 586 (41.9) |
| 20-64 | 430 (30.8) |
| ≥ 65 | 381 (27.3) |
| **Gender** | |
| Male | 957 (68.5) |
| Female | 440 (31.5) |
| **Area** | |
| North | 546 (39.1) |
| Central | 518 (37.1) |
| South | 307 (22.0) |
| East | 26 (1.9) |
| **Urbanization** | |
| Urbanized | 1019 (72.9) |
| Rural | 378 (27.1) |
| **Monthly Insurance Premiums (NTD)** | |
| <20000 | 1030 (73.7) |
| 20000-39999 | 273 (19.5) |
| ≥40000 | 94 (6.7) |

NTD, New Taiwan Dollars.

only

eastern (1.9%) areas; as high as 72.9% of the patients resided in urban areas. A majority (73.7%) of the patients had a monthly insurance premium of less than 20,000 NTD, which could be attributed to the predominance of young patients in this population.

**Incidence and mortality**

The average annual incidence of CAA from 2005 to 2011 in Taiwan was 0.87 per 100,000 population. About 200 patients were diagnosed with CAA every year. As shown in **Figure 3**, there was no significant difference in incidence from 2005 to 2011 in the trend test ($p$=0.2653).

The mortality of the CAA population was low at an average of 0.05 per 100,000 population every year; however, it increased slightly from 0.03 per 100,000 population in 2005 to 0.07 per 100,000 population in 2011 ($p$=0.0018; **Figure 3**). A total of 80 cases (17 adults and 63 elderly) died during the study period. No case in the pediatric group died; therefore, it may have attenuated the overall mortality. Specifically, the mortality rate in adults was 9.9% at a mean follow-up period of 43.3 ± 25.8 months.

**Case-control study population**

The selection process of subjects between case and control groups has been shown in **Figure 1**. Of 1397 patients identified in the previous epidemiologic study, we finally enrolled 719 adult patients with a hospitalized diagnosis of CAA as case groups. After matching by age,

For peer review only

sex, and index year, there were7190 subjects in the control group, yielding an overall population of 7909 subjects in the case-control study.

Overall, the age and sex distributions were well matched in the case and control groups (**Table 2**). The mean age of the case group was 62.57 ± 13.91 years and 67.9% were male, which was similar to the control group. There was no significant difference in the urbanization level and monthly insurance premiums. Most of the patients lived in an urban area and had a low monthly insurance premium of less than 40,000 New Taiwan Dollars.

**Risk factors associated with CAA**

In the logistic regression analysis, the association between traditional cardiovascular risk factors and the presence of CAA was higher than with the general population(**Table 2**). The adjusted odds ratio (aOR) for coronary atherosclerosis (aOR: 7.97; 95% CI: 6.46–9.84), hypertension (aOR: 2.09; 95% CI: 1.73–2.53) and dyslipidemia (aOR: 2.48; 95% CI: 2.06–2.99) were greater than two-fold in Model 1. By multivariate analysis in Model 2, we found that diabetes mellitus (aOR: 1.51; 95% CI: 1.26–1.81), aortic dissection (aOR: 6.76; 95% CI: 1.89–24.14), aortic aneurysm (aOR: 5.82; 95% CI: 2.02–16.83)and systemic lupus erythematosus (aOR: 4.09; 95% CI: 1.32–12.62)were significantly associated with the presence of CAA. After adjusting all variables in Model 3, a reverse association between

For peer review only

Table 2. Odds ratio for risk factors associated with coronary artery aneurysm

| Variables | CAA (n=719) | Control (n=7190) | Crude OR | Model 1 [a] Adjusted OR | Model 2 [b] Adjusted OR | Model 3 [c] Adjusted OR |
|---|---|---|---|---|---|---|
| **Demographics**[#] | | | | | | |
| Age | 62.57 (13.91) | 62.57 (13.92) | - | - | - | - |
| Male sex | 488 (67.9) | 4880 (67.9) | - | - | - | - |
| Urbanization (urbanized vs. rural) | 635 (88.3) | 6441 (89.6) | 1.04 (0.87-1.24) | 0.95 (0.79-1.15) | 1.02 (0.85-1.22) | 0.95 (0.78-1.15) |
| Insurance premiums (high vs. low) | 84 (11.7) | 749 (10.4) | 1.15 (0.90-1.47) | 0.93 (0.70-1.23) | 1.14 (0.89-1.46) | 0.92 (0.69-1.21) |
| **Risk factors** | | | | | | |
| Coronary atherosclerosis | 244 (33.9) | 354 (4.9) | 10.82 (8.85-13.24)* | 7.97 (6.46-9.84)* | | 8.00 (6.47-9.90)* |
| Hypertension | 459 (63.8) | 2836 (39.4) | 3.19 (2.68-3.80)* | 2.09 (1.73-2.53)* | | 2.12 (1.75-2.58)* |
| Dyslipidemia | 289 (40.2) | 1126 (15.7) | 3.77 (3.19-4.45)* | 2.48 (2.06-2.99)* | | 2.60 (2.15-3.14)* |
| Diabetes mellitus | 188 (26.1) | 1457 (20.3) | 1.51 (1.26-1.80)* | | 1.51 (1.26-1.81)* | 0.82 (0.67-1.00) |
| Cerebrovascular disease | 74 (10.3) | 710 (9.9) | 1.05 (0.81-1.36) | | 1.02 (0.78-1.32) | 0.90 (0.68-1.20) |
| Peripheral vascular disease | 4 (0.6) | 59 (0.8) | 0.68 (0.25-1.87) | | 0.66 (0.24-1.82) | 0.47 (0.15-1.44) |
| Varicose vein | 2 (0.3) | 47 (0.7) | 0.43 (0.10-1.75) | | 0.46 (0.11-1.91) | 0.41 (0.09-1.90) |
| Aortic dissection | 4 (0.6) | 6 (0.1) | 6.67 (1.88-23.62)* | | 6.76 (1.89-24.14)* | 3.78 (0.77-18.49) |
| Aortic aneurysm | 6 (0.8) | 9 (0.1) | 6.67 (2.37-18.73)* | | 5.82 (2.02-16.83)* | 3.96 (1.19-13.20)* |
| Systemic lupus erythematosus | 5 (0.7) | 10 (0.1) | 5.00 (1.71-14.63)* | | 4.09 (1.32-12.62)* | 3.26 (0.94-11.28) |
| Rheumatoid arthritis | 9 (1.3) | 48 (0.7) | 1.89 (0.92-3.86) | | 1.80 (0.87-3.73) | 1.40 (0.60-3.25) |
| Inflammatory bowel disease | 1 (0.1) | 31 (0.4) | 0.32 (0.04-2.36) | | 0.35 (0.05-2.54) | 0.49 (0.07-3.67) |

* $P < 0.05$; [#] Age and gender for matching were not analyzed in models. OR, odds ratio; Different covariates were analyzed in three models: [a] demographics plus traditional cardiovascular diseases except diabetes; [b] demographics plus diabetes and other risk factors; [c] all covariates mentioned above.

diabetes mellitus and CAA was found. For the discrepancy, we found a moderate collinearity between diabetes mellitus and cardiovascular risk factors (Supplementary **eTable 1** online); therefore, the results in Model 2 may be more reasonable to interpret and explain.

Other vascular diseases, such as cerebrovascular disease, peripheral vascular disease and varicosities, were not statistically significant risk factors for CAA. Similarly, rheumatoid arthritis and inflammatory bowel disease failed to show significant differences between the two groups.

**Discussion**

The average annual incidence of CAA from 2005 to 2011 in Taiwan was 0.87 per 100,000 population, with around 200 new cases every year. There was no increasing trend in the incidence of CAA during this period.

Based on case series on angiography, the incidence of CAA ranged from 0.2%to 6.0%,[2, 4] and was higher at 3.0% to 9.4%[25, 26] when diagnosed by computed tomography coronary angiography. Two reported case series in Taiwan on 10120 and 924 patients[5, 6] accounted for a prevalence of 0.2% and 2.6%, respectively. Despite the low incidence of CAA in our study, it may be incomparable with that of previous reports owing to different sources of the sample population; we retrieved the CAA population from the NHIRD, whereas other studies derived their data from angiography case series.

It is believed that CAA to be detected more frequently with the advent of advanced

For peer review only

imaging studies and as interventional management of coronary artery disease become more common.[27] Nonetheless, a series in northern Greece[3] showed that the prevalence of coronary artery ectasia remained unchanged during the study period from 1995 to 2003, which was in consistent with our results. A possible explanation for the low incidence of CAA in our study and other literature may be the fact that CAA is usually asymptomatic until the size becomes large enough to induce complications; only patients with ischemic symptoms and admitted for further examinations will be diagnosed.

The average annual mortality rate of patients with CAA in our study was 0.05 per 100,000 population. Based on our results, all cases of death occurred in patients >20 years; therefore, mortality may have been attenuated by the pediatric population. However, if we focused on the adult population, an overall mortality rate of 9.9% at a mean follow-up of 43.3 ± 25.8 months was noted.

The mortality of patients with CAA varies widely among different studies. The 5-year mortality reached as high as 29% in the United States, based on a case series by Baman et al.[20] Similarly, the Coronary Artery Surgery Study[1] found that patients with CAA as well as a coexistent coronary stenosis had a 26% mortality at five years. In contrast, other countries have a lower mortality rate ranging from1.4% to 10.7%.[6, 28-33] This discrepancy may be owing to a more severe obstructive coronary artery disease and longer follow-up time in the two studies. On the other hand, a small case series comprising 24 patients with CAA in

For peer review only

Taiwan[6] reported a mortality rate of 9.1%, which was similar to the results of our study despite a younger population and shorter follow-up time.

In contrast to previous angiography case series in adults, the present study had access to information on both pediatric and adult patients with CAA. The mean age of the pediatric population was 3.16 ± 3.66 years. Up to 95.7% of children were diagnosed with Kawasaki disease. Similar to other epidemiologic reports in Taiwan,[11, 34, 35] our results showed that most patients with Kawasaki disease were under 5 years of age; infants ≤1 year of age comprised the majority of this population. Kawasaki disease is a systemic vasculitis that commonly involves the coronary arteries and is considered the main cause of nonatherosclerotic coronary artery aneurysm in young children.[23, 36] From our results, the disease burden of Kawasaki disease in Taiwan and its risk for developing CAA cannot be overlooked. For young patients who present with an ischemic heart syndrome, such as angina and myocardial infarction, CAA should be considered and treatment should be started as soon as possible.

In the multivariate analysis, traditional cardiovascular risk factors, including coronary atherosclerosis, hypertension, dyslipidemia and diabetes, were independently associated with CAA.

Coronary atherosclerosis is a well-established risk factor for CAA in literature.[1, 8, 20, 36, 37] For Asian countries, a small series containing 24 CAA patients in Taiwan reported over 60% patients had multivessel disease.[6] In China, another two series showed a even higher proportion

For peer review only

of coronary artery disease in patients with CAA.[26, 38] There are various etiologies of CAA being extensively discussed, among which atherosclerosis remains the most common factor and accounts for over 50% of acquired CAA in adults.[1, 8, 23, 36] It has been hypothesized that atherosclerosis is linked to aneurysm formation through a process of inflammation extending into the tunica media from the tunica intima, which eventually causes degeneration of the cystic medial.[23, 39] For mechanic factors, the blood flow through the stenotic area in atherosclerotic vessels may cause turbulent and reversed flow, which can induce endothelial damage and increase wall stress and subsequently result in post-stenotic dilatation.[9, 40]

Some studies have reported that hypertension was more common in patients with CAA,[21, 26, 41, 42] whereas no significant difference was found in other series.[20, 22, 28, 37, 41, 43] Only one study showed a lower frequency of hypertension in patients with CAA.[6] Nichols et al[23] indicated that hypertension was related to aortic and cerebral artery aneurysms; therefore an association between hypertension and CAA may be expected. However, they drew a conclusion that there was no significant association between hypertension and CAA after reviewing the literature. On the other hand, Markis et al[44] suggested that hypertension may play a role in the development of CAA, perhaps through accelerating the process of media destruction. Another viewpoint from Yip et al,[6] they proposed that the formation of CAA may be explained by the law of LaPlace, in which the wall stress is in direct proportion to intramural pressure. In the hypertensive state, intramural pressure against the vessel wall leads

For peer review only

to dilatation of the coronary arteries.

Similarly, there were some studies showing that dyslipidemia was more prevalent in patients with CAA,[6, 20, 26, 41] in contrast to other reports.[18, 22, 28, 37, 43, 45, 46] Evidence indicates dyslipidemia is a major contributor to atherosclerosis, which is characterized by the deposition of lipids and fibrous elements in the arteries and further formation of the atheroma.[39] In view of atherosclerosis, it may be reasonable that our study showed a positive association between dyslipidemia and the presence of CAA.

It is noteworthy that an inverse association between diabetes and the presence of CAA had been reported in several studies,[18, 20, 21, 28, 46] but not in others.[31, 33, 40, 47, 48] A previous meta-analysis[49] indicated diabetes as a protective factor for the occurrence of CAA, with a pooled OR of 0.65 (95% CI, 0.54–0.77). For positive association, it was reported that hyperglycemia can trigger arterial inflammation and increase the risk of atherosclerosis,[39] which is a risk factor for CAA. Nonetheless, debate on whether CAA is a variant of atherosclerosis still exists.[50] Conversely, some authors proposed that the protective effect of diabetes may be from negative arterial remodeling[46] and increased matrix volume[49] of the coronary arteries.

As a whole, the association between diabetes and CAA is currently not well-defined. We deduce that the conflicting results may have arisen from the different selection criteria for the control group and the small sample size in some series. Most of the studies selected controls

For peer review only

from a cohort undergoing coronary angiography, whereas ours derived their subjects from the general population. The burden of cardiovascular disease on the controls may be more severe in the other series than in ours.

In the current study, we also found a significantly positive association between aortic dissection, aortic aneurysm, and systemic lupus erythematosus and the presence of CAA.

Aortic aneurysms have been reported to be prevalent in patients with CAA,[26, 42, 51] and patients with aortic aneurysms also had a higher frequency of CAA.[52-54] It had been speculated that CAA and aortic aneurysms share a similar histology and pathogenic process, which is generalized impairment of the wall of the entire arterial system.[51, 55] The matrix metalloproteinases, a group of enzymes with the capacity to degrade various components of the extracellular matrix in the arterial wall, have been found with elevated levels both in patients with CAA[56] and patients with aortic aneurysms.[57] This finding implicated that aortic and coronary aneurysms may develop through the similar process of increased destruction of extracellular matrix.

With respect to systemic lupus erythematosus, an increasing number of case reports have indicated its relationship with CAA in recent years.[58-63] In addition, systemic lupus erythematosus was found to be associated with aortic aneurysms.[64, 65] Systemic lupus erythematosus is a chronic inflammatory disease and associated with cardiac manifestations, such as pericarditis, myocarditis, valvular disease and coronary artery disease.[66] It was

For peer review only

suggested that CAA resulted from severe coronary inflammation through the deposition of immune globulin and complement.[67] At other aspects, systemic lupus erythematosus was thought to induce accelerated atherosclerosis, which was a risk factor for aneurysms.[68] Although there were reports suggesting the relationship between systemic lupus erythematosus and development of CAA, the exact pathogenesis remains poorly understood.

To the best of our knowledge, this was the first population-based study investigating the epidemiology and risk factors for CAA in Taiwan. Using a specific NHIRD dataset on all patients with CAA, we were able to provide an explicit age distribution, including both pediatric and adult populations. This was unavailable in angiographic series enrolling mainly adult subjects. Furthermore, we analyzed potential risk factors for CAA by performing a case–control study through multivariate analysis, which may disclose certain rare risk factors associated with CAA.

However, there were some limitations in the current study. First, we were unable to estimate the prevalence because not all patients in the epidemiologic study were available for follow-up. Second, our data were obtained from a claim-based database, which did not include information on aneurysm features, such as size, shape, location, number of aneurysms, and degree of stenosis. In addition, we could not differentiate between aneurysm and ectasia in this study. Third, the control group selected from the general population may have had less opportunity to undergo examinations to detect atherosclerosis and aortic aneurysm. For this

For peer review only

reason, the control group may have had fewer related events than the case group. Finally, cardiovascular risk factors of smoking and obesity were unavailable from our database.

In conclusion, the incidence of CAA in Taiwan was low and did not increase from 2005 to 2011. Kawasaki disease was predominant in children with CAA. Aside from the cardiovascular diseases, factors such as aortic disease and systemic lupus erythematosus, may predispose adults to develop CAA.

For peer review only

**Acknowledgements**

This study is based in part on data from the NHIRD provided by the Bureau of the NHI, Department of Health and managed by National Health Research Institutes. The authors acknowledge the assistance of the Bureau of the NHI and National Health Research Institutes. This work was supported by the Ministry of Science and Technology (MOST 104-2320-B-037 -035)and Kaohsiung Medical University Hospital (KMUH104-M417)

**Author contributions**

Chein-Tang Fang, Yaw-Bin Huang, and Chung-Yu Chen designed the data collection instruments, conceptualized and designed the study, drafted the initial manuscript and approved the final manuscript as submitted. Chein-Tang Fang, Yi-Ping Fang and Chung-Yu Chen carried out the initial analyses, critically reviewed the manuscript, and all authors approved the final manuscript as submitted.

**Funding:** This work was supported by grants from Ministry of Science and Technology (MOST 104-2320-B-037 -035) and Kaohsiung Medical University Hospital (KMUH104-M417)

**Competing interests:** None declared.

**Ethics approval:** This study was approved by the Institutional Review Board of Kaohsiung Medical University Hospital.

**Provenance and peer review:** Not commissioned; externally peer reviewed.

**Data sharing statement:** No additional data are available.

For peer review only

## References


1. Swaye PS, Fisher LD, Litwin P, Vignola PA, Judkins MP, Kemp HG, et al. Aneurysmal coronary artery disease. *Circulation*. 1983;67(1):134-8.
2. Tunick PA, Slater J, Kronzon I, Glassman E. Discrete atherosclerotic coronary artery aneurysms: a study of 20 patients. *J Am Coll Cardiol*. 1990;15(2):279-82.
3. Giannoglou GD, Antoniadis AP, Chatzizisis YS, Damvopoulou E, Parcharidis GE, Louridas GE. Prevalence of ectasia in human coronary arteries in patients in northern Greece referred for coronary angiography. *Am J Cardiol*. 2006;98(3):314-8.
4. Almansori MA, Elsayed HA. Coronary artery ectasia - A sample from Saudi Arabia. *J Saudi Heart Assoc*. 2015;27(3):160-3.
5. Wang KY, Ting CT, St John Sutton M, Chen YT. Coronary artery aneurysms: A 25-patient study. *Catheter Cardiovasc Interv*. 1999;48(1):31-8.
6. Yip HK, Chen MC, Wu CJ, Hang CL, Hsieh KY, Fang CY, et al. Clinical features and outcome of coronary artery aneurysm in patients with acute myocardial infarction undergoing a primary percutaneous coronary intervention. *Cardiology*. 2002;98(3):132-40.
7. Swanton RH, Thomas ML, Coltart DJ, Jenkins BS, Webb-Peploe MM, Williams BT. Coronary artery ectasia--a variant of occlusive coronary arteriosclerosis. *Br Heart J*. 1978;40(4):393-400.
8. Cohen P, O'Gara PT. Coronary artery aneurysms: a review of the natural history, pathophysiology, and management. *Cardiol Rev*. 2008;16(6):301-4.
9. Diaz-Zamudio M, Bacilio-Perez U, Herrera-Zarza MC, Meave-Gonzalez A, Alexanderson-Rosas E, Zambrana-Balta GF, et al. Coronary artery aneurysms and ectasia: role of coronary CT angiography. *Radiographics*. 2009;29(7):1939-54.
10. Uehara R, Belay ED. Epidemiology of Kawasaki disease in Asia, Europe, and the United States. *J Epidemiol*. 2012;22(2):79-85.
11. Lin MC, Lai MS, Jan SL, Fu YC. Epidemiologic features of Kawasaki disease in acute stages in Taiwan, 1997-2010: effect of different case definitions in claims data analysis. *J Chin Med Assoc*. 2015;78(2):121-6.
12. Manginas A, Cokkinos DV. Coronary artery ectasias: imaging, functional assessment and clinical implications. *Eur Heart J*. 2006;27(9):1026-31.
13. Antoniadis AP, Chatzizisis YS, Giannoglou GD. Pathogenetic mechanisms of coronary ectasia. *Int J Cardiol*. 2008;130(3):335-43.
14. Rath S, Har-Zahav Y, Battler A, Agranat O, Rotstein Z, Rabinowitz B, et al. Fate of nonobstructive aneurysmatic coronary artery disease: angiographic and clinical follow-up report. *Am Heart J*. 1985;109(4):785-91.
15. Kruger D, Stierle U, Herrmann G, Simon R, Sheikhzadeh A. Exercise-induced


For peer review only

myocardial ischemia in isolated coronary artery ectasias and aneurysms ("dilated coronopathy"). *J Am Coll Cardiol*. 1999;34(5):1461-70.

16. Sayin T, Doven O, Berkalp B, Akyurek O, Gulec S, Oral D. Exercise-induced myocardial ischemia in patients with coronary artery ectasia without obstructive coronary artery disease. *Int J Cardiol*. 2001;78(2):143-9.
17. Cheng CL, Chien HC, Lee CH, Lin SJ, Yang YH. Validity of in-hospital mortality data among patients with acute myocardial infarction or stroke in National Health Insurance Research Database in Taiwan. *Int J Cardiol*. 2015;201:96-101.
18. Androulakis AE, Andrikopoulos GK, Kartalis AN, Stougiannos PN, Katsaros AA, Syrogiannidis DN, et al. Relation of coronary artery ectasia to diabetes mellitus. *Am J Cardiol*. 2004;93(9):1165-7.
19. Androulakis AE, Katsaros AA, Kartalis AN, Stougiannos PN, Andrikopoulos GK, Triantafyllidi EI, et al. Varicose veins are common in patients with coronary artery ectasia. Just a coincidence or a systemic deficit of the vascular wall? *Eur J Vasc Endovasc Surg*. 2004;27(5):519-24.
20. Baman TS, Cole JH, Devireddy CM, Sperling LS. Risk factors and outcomes in patients with coronary artery aneurysms. *Am J Cardiol*. 2004;93(12):1549-51.
21. Saglam M, Karakaya O, Barutcu I, Esen AM, Turkmen M, Kargin R, et al. Identifying cardiovascular risk factors in a patient population with coronary artery ectasia. *Angiology*. 2007;58(6):698-703.
22. Amirzadegan AR, Davoodi G, Soleimani A, Lotfi Tokaldany M, Hakki Kazazi E, Shabpiray H, et al. Association between Traditional Risk Factors and Coronary Artery Ectasia: A Study on 10057 Angiographic Procedures among Iranian Population. *J Tehran Heart Cent*. 2014;9(1):27-32.
23. Nichols L, Lagana S, Parwani A. Coronary artery aneurysm: a review and hypothesis regarding etiology. *Arch Pathol Lab Med*. 2008;132(5):823-8.
24. Department of Household Registration, Ministry of the Interior. Midyear Population by Sex and 5-Year Age Group. http://www.ris.gov.tw/web/ris3-english/history; Accessed 16.04.10.
25. Leschka S, Stolzmann P, Scheffel H, Wildermuth S, Plass A, Genoni M, et al. Prevalence and morphology of coronary artery ectasia with dual-source CT coronary angiography. *Eur Radiol*. 2008;18(12):2776-84.
26. Yang JJ, Yang X, Chen ZY, Wang Q, He B, Du LS, et al. Prevalence of coronary artery ectasia in older adults and the relationship with epicardial fat volume by cardiac computed tomography angiography. *J Geriatr Cardiol*. 2013;10(1):10-5.
27. LaMendola CL, Culliford AT, Harris LJ, Amendo MT. Multiple aneurysms of the coronary arteries in a patient with systemic aneurysmal disease. *Ann Thorac Surg*. 1990;49(6):1009-10.

For peer review only

28. Pinar Bermudez E, Lopez Palop R, Lozano Martinez-Luengas I, Cortes Sanchez R, Carrillo Saez P, Rodriguez Carreras R, et al. Coronary ectasia: prevalence, and clinical and angiographic characteristics. *Rev Esp Cardiol*. 2003;56(5):473-9.
29. Lam CS, Ho KT. Coronary artery ectasia: a ten-year experience in a tertiary hospital in Singapore. *Ann Acad Med Singapore*. 2004;33(4):419-22.
30. Valente S, Lazzeri C, Giglioli C, Sani F, Romano SM, Margheri M, et al. Clinical expression of coronary artery ectasia. *J Cardiovasc Med (Hagerstown)*. 2007;8(10):815-20.
31. Yilmaz H, Sayar N, Yilmaz M, Tangurek B, Cakmak N, Gurkan U, et al. Coronary artery ectasia: clinical and angiographical evaluation. *Turk Kardiyol Dern Ars*. 2008;36(8):530-5.
32. Sultana R, Sultana N, Ishaq M, Samad A. The prevalence and clinical profile of angiographic coronary ectasia. *J Pak Med Assoc*. 2011;61(4):372-5.
33. Boles U, Zhao Y, Rakhit R, Shiu MF, Papachristidis A, David S, et al. Patterns of coronary artery ectasia and short-term outcome in acute myocardial infarction. *Scand Cardiovasc J*. 2014;48(3):161-6.
34. Chang LY, Chang IS, Lu CY, Chiang BL, Lee CY, Chen PJ, et al. Epidemiologic features of Kawasaki disease in Taiwan, 1996-2002. *Pediatrics*. 2004;114(6):e678-82.
35. Huang WC, Huang LM, Chang IS, Chang LY, Chiang BL, Chen PJ, et al. Epidemiologic features of Kawasaki disease in Taiwan, 2003-2006. *Pediatrics*. 2009;123(3):e401-5.
36. Pahlavan PS, Niroomand F. Coronary artery aneurysm: a review. *Clin Cardiol*. 2006;29(10):439-43.
37. Demopoulos VP, Olympios CD, Fakiolas CN, Pissimissis EG, Economides NM, Adamopoulou E, et al. The natural history of aneurysmal coronary artery disease. *Heart*. 1997;78(2):136-41.
38. Huang QJ, Zhang Y, Li XL, Li S, Guo YL, Zhu CG, et al. Clinical features of coronary artery ectasia in the elderly. *J Geriatr Cardiol*. 2014;11(3):185-91.
39. Libby P. Inflammation in atherosclerosis. *Nature*. 2002;420(6917):868-74.
40. Daoud AS, Pankin D, Tulgan H, Florentin RA. Aneurysms of the coronary artery. Report of ten cases and review of literature. *Am J Cardiol*. 1963;11:228-37.
41. Gunes Y, Boztosun B, Yildiz A, Metin Esen A, Saglam M, Bulut M, et al. Clinical profile and outcome of coronary artery ectasia. *Heart*. 2006;92(8):1159-60.
42. Farrag A, Faramawy AE, Salem MA, Wahab RA, Ghareeb S. Coronary artery ectasia diagnosed using multidetector computed tomography: morphology and relation to coronary artery calcification. *Int J Cardiovasc Imaging*. 2013;29(2):427-33.
43. Boles U, Zhao Y, David S, Eriksson P, Henein MY. Pure coronary ectasia differs from atherosclerosis: morphological and risk factors analysis. *Int J Cardiol*. 2012;155(2):321-3.

For peer review only

44. Markis JE, Joffe CD, Cohn PF, Feen DJ, Herman MV, Gorlin R. Clinical significance of coronary arterial ectasia. *Am J Cardiol*. 1976;37(2):217-22.
45. Zeina AR, Sharif D, Blinder J, Rosenschein U, Barmeir E. Noninvasive assessment of coronary artery ectasia using multidetector computed tomography. *Coron Artery Dis*. 2007;18(3):175-80.
46. Dogan A, Arslan A, Yucel H, Aksoy F, Icli A, Ozaydin M, et al. Gamma glutamyltransferase, inflammation and cardiovascular risk factors in isolated coronary artery ectasia. *Rev Port Cardiol*. 2016;35(1):33-9.
47. Waly HM, Elayda MA, Lee VV, el-Said G, Reul GJ, Hall RJ. Coronary artery ectasia in Egyptian patients with coronary artery disease. *Tex Heart Inst J*. 1997;24(4):349-52.
48. Sen N, Ozcan F, Uygur B, Aksu T, Akpinar I, Cay S, et al. Elevated serum uric acid levels in patients with isolated coronary artery ectasia. *Turk Kardiyol Dern Ars*. 2009;37(7):467-72.
49. Huang QJ, Liu J, Chen MH, Li JJ. Relation of diabetes to coronary artery ectasia: A meta-analysis study. *Anadolu Kardiyol Derg*. 2014;14(4):322-7.
50. Yetkin E, Waltenberger J. Novel insights into an old controversy: is coronary artery ectasia a variant of coronary atherosclerosis? *Clin Res Cardiol*. 2007;96(6):331-9.
51. Lamblin N, Bauters C, Hermant X, Lablanche JM, Helbecque N, Amouyel P. Polymorphisms in the promoter regions of MMP-2, MMP-3, MMP-9 and MMP-12 genes as determinants of aneurysmal coronary artery disease. *J Am Coll Cardiol*. 2002;40(1):43-8.
52. Kishi K, Ito S, Hiasa Y. Risk factors and incidence of coronary artery lesions in patients with abdominal aortic aneurysms. *Intern Med*. 1997;36(6):384-8.
53. Papadakis MC, Leontiadis E, Manginas A, Voudris V, Pavlides G, Karatasakis G, et al. Frequency of coronary artery ectasia in patients undergoing surgery for ascending aortic aneurysms. *Am J Cardiol*. 2004;94(11):1433-5.
54. Balderston JR, Giri J, Kolansky DM, Bavaria JE, Gertz ZM. Coronary artery aneurysms associated with ascending aortic aneurysms and abdominal aortic aneurysms: pathophysiologic implications. *Catheter Cardiovasc Interv*. 2015;85(6):961-7.
55. Stajduhar KC, Laird JR, Rogan KM, Wortham DC. Coronary arterial ectasia: increased prevalence in patients with abdominal aortic aneurysm as compared to occlusive atherosclerotic peripheral vascular disease. *Am Heart J*. 1993;125(1):86-92.
56. Tengiz I, Ercan E, Aliyev E, Sekuri C, Duman C, Altuglu I. Elevated levels of matrix metalloprotein-3 in patients with coronary aneurysm: A case control study. *Curr Control Trials Cardiovasc Med*. 2004;5(1):10.
57. Carrell TW, Burnand KG, Wells GM, Clements JM, Smith A. Stromelysin-1 (matrix metalloproteinase-3) and tissue inhibitor of metalloproteinase-3 are overexpressed in the wall of abdominal aortic aneurysms. *Circulation*. 2002;105(4):477-82.

For peer review only

58. Yoshikai M, Hamada M, Takarabe K. Coronary artery aneurysm with systemic lupus erythematosus. *Jpn J Thorac Cardiovasc Surg*. 2004;52(8):379-82.
59. Suzuki H, Fujigaki Y, Mori M, Yamamoto T, Kato A, Wakahara N, et al. Giant coronary aneurysm in a patient with systemic lupus erythematosus. *Intern Med*. 2009;48(16):1407-12.
60. Nagao T, Ito K, Tsuboi H, Ide Y. Rare complications of systemic lupus erythematosus. *Intern Med*. 2012;51(21):3101-2.
61. Posadzy-Malaczynska A, Woznicka-Leskiewicz L, Juszkat R, Frankiewicz M, Kociemba A. Right coronary artery aneurysm with fistula into the coronary sinus in patient with systemic lupus erythematosus. *Kardiol Pol*. 2013;71(12):1329.
62. Chandra R, Avila MD, Beohar N. A rare case of lupus with multiple unusual cardiovascular complications. *Eur Heart J*. 2014;35(45):3193.
63. Monigari N, Poondru RR, Shetty RK, Vivek G. Giant coronary aneurysm presenting as acute coronary syndrome in a patient with systemic lupus erythematosus. *BMJ Case Rep*. 2014;2014.
64. Wang SH, Chang YS, Liu CJ, Lai CC, Chen TJ, Chen WS. Incidence and risk analysis of aortic aneurysm and aortic dissection among patients with systemic lupus erythematosus: a nationwide population-based study in Taiwan. *Lupus*. 2014;23(7):665-71.
65. Guy A, Tiosano S, Comaneshter D, Tekes-Manova D, Shovman O, Cohen AD, et al. Aortic aneurysm association with SLE - a case-control study. *Lupus*. 2016.
66. Miner JJ, Kim AH. Cardiac manifestations of systemic lupus erythematosus. *Rheum Dis Clin North Am*. 2014;40(1):51-60.
67. Famularo G, Vallone A, Compagnucci M, Capo G, Minisola G, Gasbarrone L. Tiny bubbles: coronary aneurysms and lupus. *Am J Med*. 2014;127(10):933-5.
68. Stojan G, Petri M. Atherosclerosis in systemic lupus erythematosus. *J Cardiovasc Pharmacol*. 2013;62(3):255-62.

For peer review only

## Figure Legends

Figure 1.Flow chart of the study population.

Figure 2.Age distribution of the CAA population

Figure 3. (a) Incidence of CAA population during 2005-2011.(b) Mortality of CAA population during 2005-2011

For peer review only

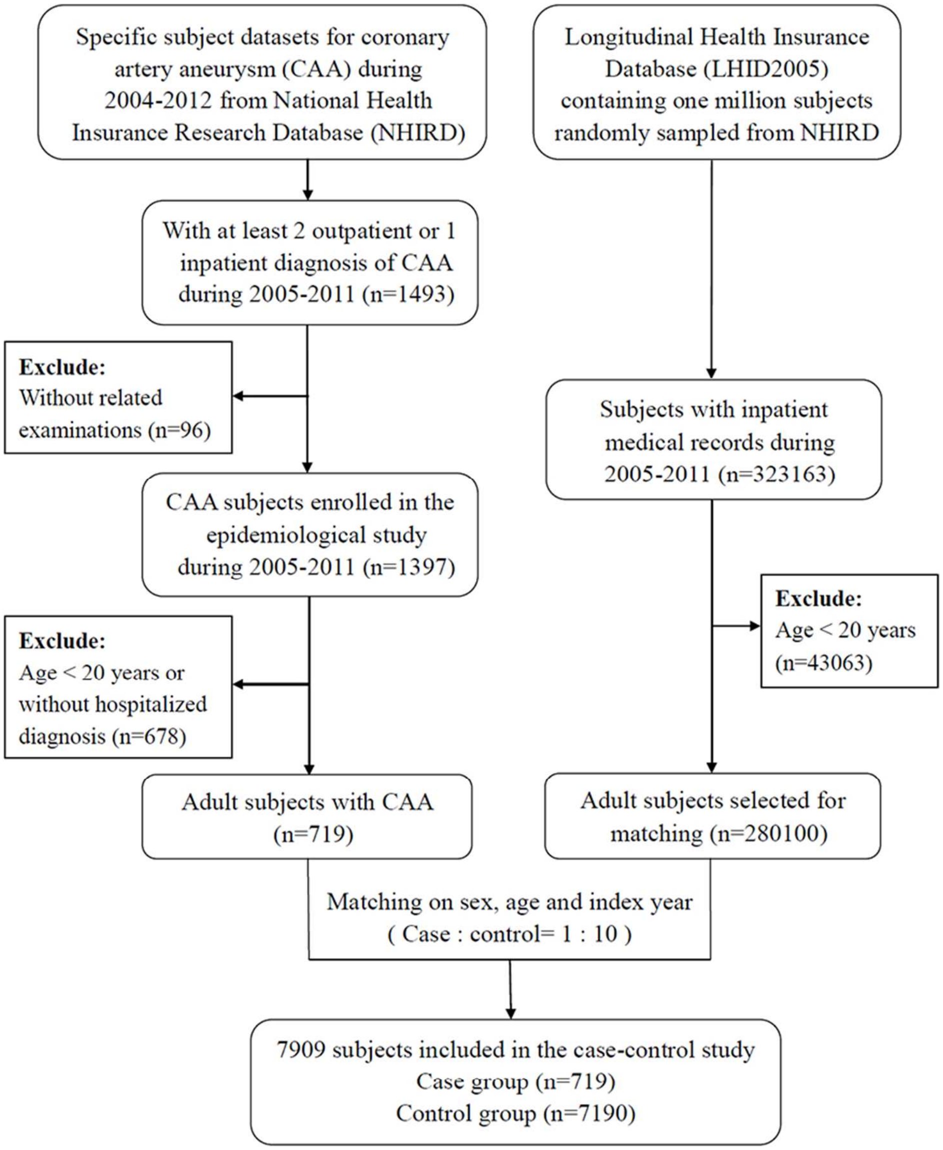


78x94mm (300 x 300 DPI)

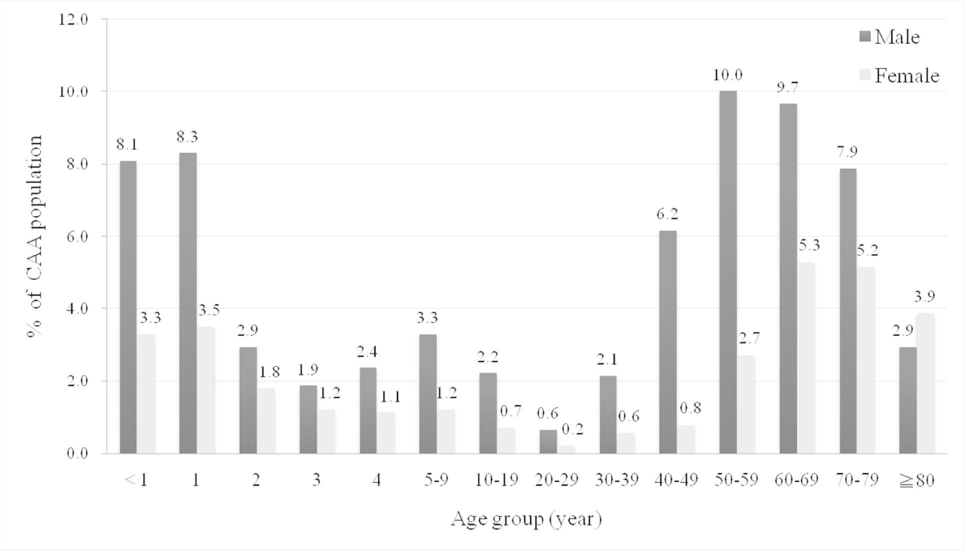


194x112mm (300 x 300 DPI)

review only

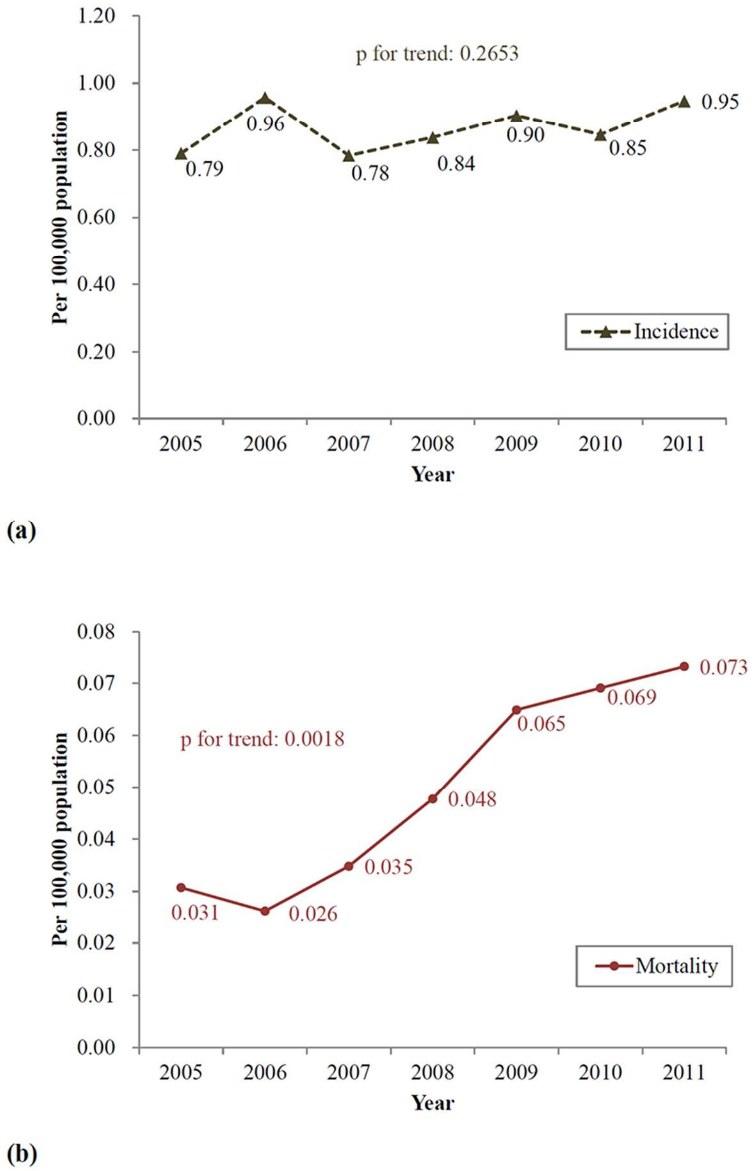


69x106mm (300 x 300 DPI)

**STROBE 2007 (v4) Statement—Checklist of items that should be included in reports of *cohort studies***

| Section/Topic | Item # | Recommendation | Reported on page # |
|---|---|---|---|
| **Title and abstract** | 1 | (*a*) Indicate the study's design with a commonly used term in the title or the abstract | 2 |
| | | (*b*) Provide in the abstract an informative and balanced summary of what was done and what was found | 2 |
| **Introduction** | | | |
| Background/rationale | 2 | Explain the scientific background and rationale for the investigation being reported | 4 |
| Objectives | 3 | State specific objectives, including any prespecified hypotheses | 4 |
| **Methods** | | | |
| Study design | 4 | Present key elements of study design early in the paper | 5-7 |
| Setting | 5 | Describe the setting, locations, and relevant dates, including periods of recruitment, exposure, follow-up, and data collection | 5-7 |
| Participants | 6 | (*a*) Give the eligibility criteria, and the sources and methods of selection of participants. Describe methods of follow-up | 5-6 |
| | | (*b*) For matched studies, give matching criteria and number of exposed and unexposed | 5-6 |
| Variables | 7 | Clearly define all outcomes, exposures, predictors, potential confounders, and effect modifiers. Give diagnostic criteria, if applicable | 5-7 |
| Data sources/ measurement | 8* | For each variable of interest, give sources of data and details of methods of assessment (measurement). Describe comparability of assessment methods if there is more than one group | 5 |
| Bias | 9 | Describe any efforts to address potential sources of bias | 5-6 |
| Study size | 10 | Explain how the study size was arrived at | 5-6 |
| Quantitative variables | 11 | Explain how quantitative variables were handled in the analyses. If applicable, describe which groupings were chosen and why | 5 |
| Statistical methods | 12 | (*a*) Describe all statistical methods, including those used to control for confounding | 8 |
| | | (*b*) Describe any methods used to examine subgroups and interactions | 8 |
| | | (*c*) Explain how missing data were addressed | 8 |
| | | (*d*) If applicable, explain how loss to follow-up was addressed | 8 |
| | | (*e*) Describe any sensitivity analyses | 8 |
| **Results** | | | |

For peer review only

| | | | |
|---|---|---|---|
| Participants | 13* | (a) Report numbers of individuals at each stage of study—eg numbers potentially eligible, examined for eligibility, confirmed eligible, included in the study, completing follow-up, and analysed | 10-13 |
| | | (b) Give reasons for non-participation at each stage | 10-13 |
| | | (c) Consider use of a flow diagram | 10 |
| Descriptive data | 14* | (a) Give characteristics of study participants (eg demographic, clinical, social) and information on exposures and potential confounders | 10 |
| | | (b) Indicate number of participants with missing data for each variable of interest | 110 |
| | | (c) Summarise follow-up time (eg, average and total amount) | 10 |
| Outcome data | 15* | Report numbers of outcome events or summary measures over time | 10-13 |
| Main results | 16 | (*a*) Give unadjusted estimates and, if applicable, confounder-adjusted estimates and their precision (eg, 95% confidence interval). Make clear which confounders were adjusted for and why they were included | 10-13 |
| | | (*b*) Report category boundaries when continuous variables were categorized | 10-13 |
| | | (*c*) If relevant, consider translating estimates of relative risk into absolute risk for a meaningful time period | 10-13 |
| Other analyses | 17 | Report other analyses done—eg analyses of subgroups and interactions, and sensitivity analyses | 10-13 |
| **Discussion** | | | |
| Key results | 18 | Summarise key results with reference to study objectives | 13 |
| **Limitations** | | | 19-20 |
| Interpretation | 20 | Give a cautious overall interpretation of results considering objectives, limitations, multiplicity of analyses, results from similar studies, and other relevant evidence | 16-18 |
| Generalisability | 21 | Discuss the generalisability (external validity) of the study results | 16-18 |
| **Other information** | | | |
| Funding | 22 | Give the source of funding and the role of the funders for the present study and, if applicable, for the original study on which the present article is based | 21 |

*Give information separately for cases and controls in case-control studies and, if applicable, for exposed and unexposed groups in cohort and cross-sectional studies.

**Note:** An Explanation and Elaboration article discusses each checklist item and gives methodological background and published examples of transparent reporting. The STROBE checklist is best used in conjunction with this article (freely available on the Web sites of PLoS Medicine at http://www.plosmedicine.org/, Annals of Internal Medicine at http://www.annals.org/, and Epidemiology at http://www.epidem.com/). Information on the STROBE Initiative is available at www.strobe-statement.org.

For peer review only

BMJ Open

# Epidemiology and risk factors of coronary artery aneurysm in Taiwan: A population-based case-control study

| | |
|---|---|
| Journal: | *BMJ Open* |
| Manuscript ID | bmjopen-2016-014424.R1 |
| Article Type: | Research |
| Date Submitted by the Author: | 01-May-2017 |
| Complete List of Authors: | Fang, Chein-Tang ; Kaohsiung Medical University<br>Fang , Yi-Ping ; Kaohsiung Medical University<br>Huang, Yaw-Bin; Kaohsiung Medical University, School of Pharmacy<br>Kuo, Chen-Chun<br>CHEN, CHUNG-YU |
| <b>Primary Subject Heading</b>: | Epidemiology |
| Secondary Subject Heading: | Cardiovascular medicine |
| Keywords: | Coronary artery aneurysm, Epidemiology < TROPICAL MEDICINE, risk factor |
| | |

SCHOLARONE™
Manuscripts

For peer review only

For peer review only - http://bmjopen.bmj.com/site/about/guidelines.xhtml

# Epidemiology and risk factors of coronary artery aneurysm in Taiwan: A population-based case-control study

Chein-Tang Fang [1], Yi-Ping Fang [1], Yaw-Bin Huang[1, 2], Chen-Chun Kuo[2], Chung-Yu Chen[1, 2]

1. School of Pharmacy, Master Program in Clinical Pharmacy, Kaohsiung Medical University, Kaohsiung, Taiwan.
2. Department of Pharmacy, Kaohsiung Medical University Hospital, Kaohsiung, Taiwan.

*Corresponding author: Chung-Yu Chen, School of Pharmacy,
No. 100, Shih-Chuan 1st Rd., Sanmin District, Kaohsiung City 80708, Taiwan (R.O.C.)
Tel: 886-7-3121101 ext 2375
Fax: 886-7-3210683
E-mail: jk2975525@hotmail.com
*Corresponding author: Yaw-Bin Huang, School of Pharmacy,
No. 100, Shih-Chuan 1st Rd., Sanmin District, Kaohsiung City 80708, Taiwan (R.O.C.)
Tel: 886-7-3121101 ext 2166
Fax: 886-7-3210683
E-mail: yabihu@kmu.edu.tw

**Word count:** 3400

**Key words**: Coronary artery aneurysm**;** epidemiology; risk factor

For peer review only

## Abstract

**Objectives:** Coronary artery aneurysm (CAA) was a usually asymptomatic and rare disease. There is limited epidemiological data of CAA in Asian or world.

**Design:** A retrospective case-control study.

**Setting:** A population-based database study from the Taiwan's National Health Insurance Research Database, included participants between 2005 and 2011.

**Participants:** CAA patients by using ICD-9-CM code: 414.11 with CAA examinations.

**Outcome measures:** The incidence rate and mortality rate of CAA were calculated. Furthermore, we also conducted matching non-CAA patients according to age, gender, index year at a 1:10 ratio to explore risk factors for CAA by conditional logistic regression.

**Result:** A total of 1397 CAA patients were identified during 2005 to 2011, including 41.9 % pediatrics and 58.1% adults. The incidence rate and mortality rate of CAA in Taiwan were 0.87 and 0.05 per $10^5$ person-years. The adjusted odds ratios (aOR) for coronary atherosclerosis, hypertension, dyslipidemia and diabetes were 7.97, 2.09, 2.48 and 1.51, respectively. Of note, aortic dissection (aOR: 6.76), aortic aneurysm (aOR: 5.82) and systemic lupus erythematosus (aOR: 4.09) were found significantly associated with CAA.

**Conclusion:** In Taiwan, CAA patients were distributed both in pediatric and adult population. Aside from cardiovascular risk factors, aortic diseases and systemic lupus erythematosus need to be further investigated in CAA patients.




For peer review only - http://bmjopen.bmj.com/site/about/guidelines.xhtml

## Strengths and limitations of this study

1. In this nationwide epidemiology study involving retrospective analysis of a claims database, we evaluated the epidemiology and risk factors for CAA in Asian country.
2. The major limitation of the study was no laboratory data on coronary angiography data and lifestyle habits of the patients.
3. As a retrospective observational analysis, this study only provides associative information from case-control study.

For peer review only



For peer review only - http://bmjopen.bmj.com/site/about/guidelines.xhtml

## Introduction

Coronary artery aneurysm (CAA) is a rare finding in patients referred for coronary angiography. It is characterized by abnormal dilatation of the vessel lumen defined as a lumen with diameter that exceeds more than 1.5-fold the diameter of the normal vessel lumens adjacent to it or as the largest coronary vessel lumen.[1] Previous case series on angiography have reported a wide range of prevalence from 0.2% to 6.0% in the wrold.[1-4] In Taiwan, Wang et al. reported 25 patients had CAA (0.25%) in 10120 patient cohort. [5] Furthermore, one case series indicated that there were 24 patients (2.6%) had aneurysmal dilatation in 924 AMI patients undergoing PCI from 1993 to 2001.[6]

Although CAA commonly coexists with atherosclerosis,[1, 7] various potential causes such as congenital, inflammatory and connective tissue disorders, and other factors have been noted in the literature.[8, 9] Among these risk factors, Kawasaki disease is well recognized as the main cause in children; it occurs predominantly in Asian countries.[10, 11] The pathogenesis of CAA is postulated to be the degradation of the extracellular matrix of the media by matrix metalloproteinases and is considered to be similar to that of other aneurysms in larger vessels.[12, 13] Instead of presenting as a benign entity, CAA shows the clinical manifestations of coronary artery diseases such as angina and acute coronary syndrome.[14-16]

Despite Kawasaki disease is a significant risk factor for the development of CAA, especially for children, information regarding CAA and its related risk factors in adult population is limited in Asian countries. Accordingly, our aim was to investigate the



For peer review only - http://bmjopen.bmj.com/site/about/guidelines.xhtml

epidemiology and risk factors for CAA in a Taiwanese population.

## Methods

### Data sources

We obtained specific dataset of all CAA patients from Taiwan's National Health Insurance Research Database (NHIRD). All medical records comprising outpatient care, inpatient care, emergency care, and prescriptions from 2005 to 2011 were used for analysis in the epidemiologic study.

For the case-control study, we selected controls from the Longitudinal Health Insurance Database (LHID2005), which contained one million samples that were randomly selected from the NHIRD and were representative of 23 million people in Taiwan. This study was approved by the Institutional Review Board of Kaohsiung Medical University Hospital (KMUH-IRB-EXEMPT-20130199). NHIRD data were de-identified by scrambling the identification codes of both patients and medical facilities, and released to the public for research purposes. Therefore, current NHIRD and hospital regulations and guidelines did not mandate informed consent in this retrospective case-control study due to we used anonymous nature of the database. All procedures performed were in accordance with the ethical standards of the institutional research committee and the directives of the Declaration of Helsinki.

### Study population

To identify patients diagnosed with CAA, we used the International Classification of



For peer review only - http://bmjopen.bmj.com/site/about/guidelines.xhtml

Diseases, Ninth Revision, Clinical Modification (ICD-9-CM) code 414.11. Patients who had two outpatient diagnoses or one inpatient diagnosis of CAA between January 1, 2005 and December 31, 2011 were identified. The index date was defined as the date of first diagnosis of CAA. Patients who were diagnosed with CAA based on imaging studies, including coronary angiography, echocardiography, computed tomography, magnetic resonance imaging and cardiac catheterization, within 90 days (3 months) before or after the index date were enrolled.

In the case-control study, cases included adult subjects who were hospitalized with a diagnosis of CAA between January 1, 2005 and December 31, 2011. Patients younger than 20 years and those who were diagnosed with CAA in the outpatient clinic were excluded. In the case group, the first hospitalization date for CAA was assigned as the index date. For the control selection, 10 patients were randomly selected for each case by matching age, sex, and index year of case diagnosis. The first admission date within the same year of CAA diagnosis was assigned as the index date for the control group.

**Survival status**

According to the validation by Cheng et al. of in-hospital mortality in the NHIRD,[17] we defined death as "4" or "A" status record upon discharge and "disenrollment" insurance status in the Registry for Beneficiaries. On the other hand, patients admitted because of an emergent reason, but without follow-up data were considered as death, which was also confirmed by checking the insurance status.



For peer review only - http://bmjopen.bmj.com/site/about/guidelines.xhtml

**Covariates**

In the epidemiologic study, we analyzed the age and sex, characteristics of subjects with respect to geographical region, urbanization, and income group at baseline. Income group was defined by the individual average monthly income during a year period before index date and classified as low (< NTD$20,000 or < US$625) and high (≥ NT$20,000 or ≥ US$625). In the case–control study, covariates for analysis included demographics and related risk factors. On the basis of previous evidences, related risk factors included coronary atherosclerosis,[8, 18, 19] hypertension,[18-21] dyslipidemia,[19-21] diabetes mellitus,[19-22] cerebrovascular disease,[8] peripheral vascular disease,[8] varicose vein,[18, 23] aortic dissection,[24] aortic aneurysm (AA),[24, 25] systemic lupus erythematosus (SLE),[8] rheumatoid arthritis,[8] inflammatory bowel disease.[8] Furthermore, we defined atherosclerosis, hypertension, and hyperlipidemia as traditional cardiovascular risk factors.[20, 21] A period of one year before the index date was reserved for covariate evaluation in both groups.

**Statistical analysis**

Incidence rate, which was presented as number per $10^5$ person-years, was defined as the number of patients with a newly-diagnosed CAA divided by the total Taiwanese population in each year. Mortality rate, which was presented as number per $10^5$ person-years, was defined as the number of patients who died divided by the total Taiwanese population in each year. For trend analysis, the Cochran–Armitage test was used to detect any significant change in



For peer review only - http://bmjopen.bmj.com/site/about/guidelines.xhtml

incidence rate or mortality rate during the study period. The demographic data of total population in Taiwan were obtained from the Statistics, Department of Household Registration, Ministry of the Interior[26]. For descriptive statistics, continuous variables were presented as mean ± SD; categorical variables were presented as frequency and proportion (%).

The odds ratio and 95% confidence interval (95% CI) for the CAA were estimated by conditional logistic regression analysis for matched pair's data and were adjusted for the study covariates. The results were expressed as three models adjusted for different variables: Model 1, demographics and traditional cardiovascular risk factors; Model 2, demographics and related risk factors, excluding traditional cardiovascular risk factors; and Model 3, demographics and related risk factors. Spearman's correlation coefficient was used to detect collinearity among variables. Data processing and statistical analysis were performed with SAS version 9.4 (SAS Inc., Cary, NC).

## Results

### Epidemiologic study population

From 2005 to 2011, there were 1493 patients who had at least two CAA diagnoses at an outpatient setting or one CAA diagnosis at an inpatient setting. After excluding 96 subjects who did not have any record of related examinations, a total of 1397 patients with CAA diagnosis were enrolled in our study (**Figure 1**). Among the 1397 patients, mean age was 37.76 ± 31.45 years. There were 586 pediatric patients (age <20 years), 430 adults (age≥20 to <65



For peer review only - http://bmjopen.bmj.com/site/about/guidelines.xhtml

For peer review only

years), and 381 elderly patients (age ≥65 years), accounting for 41.9%, 30.8%, and 27.3% of the CAA population, respectively (**Table 1**). **Figure 2** shows the detailed age distribution, where in majority of pediatric patients were < 5 years of age; in particular, patients who were ≤ 1 year of age comprised 229 patients (23.2%). Furthermore, a male predilection (68.5%) was found, with a male to female ratio of 2.18. Most of the patients with CAA lived in northern Taiwan (39.1%), followed by central (37.1%), southern (22.0%), and eastern (1.9%) areas; as high as 72.9% of the patients resided in urban areas. A majority (73.7%) of the patients were low income group, which could be attributed to the predominance of young patients in this population.

**Incidence rate and mortality rate**

The incidence rate of CAA from 2005 to 2011 in Taiwan was 0.87 per $10^5$ person-years. About 200 patients were diagnosed with CAA every year. As shown in **Figure 3**, there was no significant difference in incidence rate from 2005 to 2011 in the trend test ($p$=0.2653).

The mortality rate of the CAA population was 0.05 per $10^5$ person-years. It increased slightly from 0.03 per $10^5$ person-years in 2005 to 0.07 per $10^5$ person-years in 2011 ($p$=0.0018; **Figure 3**). A total of 80 cases (17 adults and 63 elderly) died during the study period. No case in the pediatric group died; therefore, it may have attenuated the overall mortality. Specifically, the mortality in 811 adults were 9.9% (80 cases) at a mean follow-up period of 43.3 ± 25.8 months.



For peer review only - http://bmjopen.bmj.com/site/about/guidelines.xhtml

Table 1. Baseline characteristics of CAA population

| Variable (N=1397) | Number (%) |
|---|---|
| **Age, year (mean ± SD)** | 37.76 ± 31.45 |
| **Age group (year)** | |
| < 20 | 586 (41.9) |
| 20-64 | 430 (30.8) |
| ≥65 | 381 (27.3) |
| **Gender** | |
| Male | 957 (68.5) |
| Female | 440 (31.5) |
| **Area** | |
| North | 546 (39.1) |
| Central | 518 (37.1) |
| South | 307 (22.0) |
| East | 26 (1.9) |
| **Urbanization** | |
| Urbanized | 1019 (72.9) |
| Rural | 378 (27.1) |
| **Income group*** | |
| Low | 1030 (73.7) |
| High | 367 (26.3) |

* Income group was defined by the individual average monthly income during a year period before index date and classified as low (< NTD$20,000) and high (≥ NT$20,000).



For peer review only - http://bmjopen.bmj.com/site/about/guidelines.xhtml

**Case-control study population**

The selection process of subjects between case and control groups has been shown in **Figure 1**. Of 1397 patients identified in the previous epidemiologic study, we finally enrolled 719 adult patients with a hospitalized diagnosis of CAA as case groups. After matching by age, sex, and index year, there were 7190 subjects in the control group, yielding an overall population of 7909 subjects in the case-control study.

Overall, the age and sex distributions were well balanced between the case and control groups (**Table 2**). The mean age of the case group was 62.57 ± 13.91 years and 67.9% were male, which was similar to the control group. There was no significant difference in the urbanization level and income group. Most of the patients lived in an urban area and low group.

**Risk factors associated with CAA**

In conditional logistic regression analysis, association between traditional cardiovascular risk factors and the presence of CAA was higher than with the general population (**Table 2**). The adjusted odds ratio (aOR) for coronary atherosclerosis (aOR: 7.97; 95% CI: 6.46–9.84), hypertension (aOR: 2.09; 95% CI: 1.73–2.53) and dyslipidemia (aOR: 2.48; 95% CI: 2.06–2.99) were greater than two-fold in Model 1. By multivariate analysis in Model 2, we found that diabetes mellitus (aOR: 1.51; 95% CI: 1.26–1.81), aortic dissection (aOR: 6.76; 95% CI: 1.89–24.14), AA (aOR: 5.82; 95% CI: 2.02–16.83) and systemic lupus



For peer review only - http://bmjopen.bmj.com/site/about/guidelines.xhtml

For peer review only

Table 2. Odds ratio for risk factors associated with coronary artery aneurysm

| Variables | CAA (n=719) | Control (n=7190) | Crude OR | Model 1 [a] Adjusted OR | Model 2 [b] Adjusted OR | Model 3 [c] Adjusted OR |
|---|---|---|---|---|---|---|
| **Demographics**[#] | | | | | | |
| Age | 62.57 (13.91) | 62.57 (13.92) | - | - | - | - |
| Male sex | 488 (67.9) | 4880 (67.9) | - | - | - | - |
| Urbanization | | | | | | |
| rural | 191 (26.6) | 1964 (27.3) | 1 | 1 | 1 | 1 |
| urbanized | 528 (73.4) | 5226 (72.7) | 1.04 (0.87-1.24) | 0.95 (0.79-1.15) | 1.02 (0.85-1.22) | 0.95 (0.78-1.15) |
| Income group[&] | | | | | | |
| low | 635 (88.3) | 6441 (89.6) | 1 | 1 | 1 | 1 |
| high | 84 (11.7) | 749 (10.4) | 1.15 (0.90-1.47) | 0.93 (0.70-1.23) | 1.14 (0.89-1.46) | 0.92 (0.69-1.21) |
| **Risk factors** | | | | | | |
| Coronary atherosclerosis | 244 (33.9) | 354 (4.9) | 10.82 (8.85-13.24)* | 7.97 (6.46-9.84)* | | 8.00 (6.47-9.90)* |
| Hypertension | 459 (63.8) | 2836 (39.4) | 3.19 (2.68-3.80)* | 2.09 (1.73-2.53)* | | 2.12 (1.75-2.58)* |
| Dyslipidemia | 289 (40.2) | 1126 (15.7) | 3.77 (3.19-4.45)* | 2.48 (2.06-2.99)* | | 2.60 (2.15-3.14)* |
| Diabetes mellitus | 188 (26.1) | 1457 (20.3) | 1.51 (1.26-1.80)* | | 1.51 (1.26-1.81)* | 0.82 (0.67-1.00) |
| Cerebrovascular disease | 74 (10.3) | 710 (9.9) | 1.05 (0.81-1.36) | | 1.02 (0.78-1.32) | 0.90 (0.68-1.20) |
| Peripheral vascular disease | 4 (0.6) | 59 (0.8) | 0.68 (0.25-1.87) | | 0.66 (0.24-1.82) | 0.47 (0.15-1.44) |
| Varicose vein | 2 (0.3) | 47 (0.7) | 0.43 (0.10-1.75) | | 0.46 (0.11-1.91) | 0.41 (0.09-1.90) |
| Aortic dissection | 4 (0.6) | 6 (0.1) | 6.67 (1.88-23.62)* | | 6.76 (1.89-24.14)* | 3.78 (0.77-18.49) |
| Aortic aneurysm | 6 (0.8) | 9 (0.1) | 6.67 (2.37-18.73)* | | 5.82 (2.02-16.83)* | 3.96 (1.19-13.20)* |
| Systemic lupus erythematosus | 5 (0.7) | 10 (0.1) | 5.00 (1.71-14.63)* | | 4.09 (1.32-12.62)* | 3.26 (0.94-11.28) |
| Rheumatoid arthritis | 9 (1.3) | 48 (0.7) | 1.89 (0.92-3.86) | | 1.80 (0.87-3.73) | 1.40 (0.60-3.25) |
| Inflammatory bowel disease | 1 (0.1) | 31 (0.4) | 0.32 (0.04-2.36) | | 0.35 (0.05-2.54) | 0.49 (0.07-3.67) |

*$P < 0.05$; [#] Age and gender for matching were not analyzed in models. OR, odds ratio; Different covariates were analyzed in three models:
[a] demographics and traditional cardiovascular risk factors; [b] demographics, and related risk factors, excluding traditional cardiovascular risk factors; [c] all covariates;
[&] Income group was defined by the individual average monthly income during a year period before index date and classified as low (< NTD$20,000) and high (≥ NT$20,000).



For peer review only - http://bmjopen.bmj.com/site/about/guidelines.xhtml

erythematosus (aOR: 4.09; 95% CI: 1.32–12.62) were significantly associated with the presence of CAA. In Model 3, traditional cardiovascular risk factors and AA were significantly associated with the presence of CAA. For the discrepancy, we found a moderate collinearity between diabetes mellitus and traditional cardiovascular risk factors (Supplementary eTable 1 online).

Other vascular diseases, such as cerebrovascular disease, peripheral vascular disease and varicosities, were not statistically significant risk factors for CAA. Similarly, rheumatoid arthritis and inflammatory bowel disease failed to show significant differences between the two groups.

For peer review only



For peer review only - http://bmjopen.bmj.com/site/about/guidelines.xhtml

## Discussion

The incidence rate of CAA from 2005 to 2011 in Taiwan was 0.87 per $10^5$ person-years, with around 200 new cases every year. There was no increasing trend in the incidence rate of CAA during this period. In the United States, there were two studies on angiography reporting CAA prevalence of 0.2% (20/8422) between July 1975 and May 1979, and 4.9% (978/20087) from July 1981 to February 1987.[1-2] A report performed in Greece for coronary angiography showed CAA prevalence of 2.7% (287/10524) from 1995 to 2003.[3] The another case series conducted from 2011 to 2013 in Saudi Arabia found 1115 patients with invasive coronary angiogram had 67 (6%) coronary artery ectasia cases.[4] Furthermore, in China, coronary artery ectasia were identified in 131 of the 1400 older adults (prevalence: 9.4%) when diagnosed by computed tomography coronary angiography.[27] Two case series in Taiwan on 10120 and 924 patients accounted for a prevalence of 0.2% (25 cases) and 2.6% (24 cases), respectively.[5, 6] Comparing with other studies derived data from angiography case series, this study retrieved CAA population from the NHIRD. Therefore, lower incidence rate was found in our study may be the fact that CAA is usually asymptomatic until the size becomes large enough to induce complications; only patients with ischemic symptoms and admitted for further examinations will be diagnosed.

The mortality rate of the CAA population was 0.05 per $10^5$ person-years and all cases of death occurred in patients >20 years. For 811 adult population, an overall mortality of 9.9%



For peer review only - http://bmjopen.bmj.com/site/about/guidelines.xhtml

(80 cases) was noted in our study. The mortality of patients with CAA varies widely among different studies. The 5-year mortality reached as high as 29% based on a case series in the United States.[19] Similarly, the Coronary Artery Surgery Study[1] found that patients with CAA as well as a coexistent coronary stenosis had a 26% mortality at five years. In contrast, other countries had a lower mortality rate ranging from 1.4% to 10.7%.[28-30] This discrepancy may be owing to a more severe obstructive coronary artery disease and longer follow-up time in the prior two studies. On the other hand, a small case series comprising 24 patients with CAA in Taiwan[6] reported a mortality rate of 9.1%, which was similar to the results of our study despite a younger population and shorter follow-up time.

In contrast to previous angiography case series in adults, the present study had access to information on both pediatric and adult patients with CAA. The mean age of the pediatric population was 3.16 ± 3.66 years. Up to 95.7% of children were diagnosed with Kawasaki disease. Similar to other epidemiologic reports in Taiwan,[11, 31, 32] our results showed that most patients with Kawasaki disease were under 5 years of age; infants ≤1 year of age comprised the majority of this population. Kawasaki disease is a systemic vasculitis that commonly involves the coronary arteries and is considered the main cause of non-atherosclerotic coronary artery aneurysm in young children.[18, 33]

In the present study, traditional cardiovascular risk factors were independently associated with CAA. Coronary atherosclerosis is a well-established risk factor for CAA in



For peer review only - http://bmjopen.bmj.com/site/about/guidelines.xhtml

literature.[1, 8, 18] A small series containing 24 CAA patients in Taiwan reported over 60% patients had multi-vessel disease.[6] There are various etiologies of CAA being extensively discussed, among which atherosclerosis remains the most common factor and accounts for 50% of acquired CAA in adults.[18, 33] It has been hypothesized that atherosclerosis is linked to aneurysm formation through a process of inflammation extending into the tunica media, which eventually causes degeneration of the cystic medial.[18]

Some studies have reported that hypertension was more common in patients with CAA,[20, 34] whereas no significant difference was found in other series.[19, 21] Only one study showed a lower frequency of hypertension in patients with CAA.[6] Nichols et al[18] indicated that hypertension was related to aortic and cerebral artery aneurysms; therefore an association between hypertension and CAA might be expected. In addition, Markis et al[35] suggested that hypertension may play a role in the development of CAA, perhaps through accelerating the process of media destruction. Evidence indicates dyslipidemia is a major contributor to atherosclerosis, which is characterized by the deposition of lipids and fibrous elements in the arteries and further formation of the atheroma.[36] In view of atherosclerosis, it may be reasonable that our study showed a positive association between dyslipidemia and the presence of CAA.

It is noteworthy that an inverse association between diabetes and the presence of CAA had been reported in several studies.[19, 20, 22] A previous meta-analysis[37] indicated diabetes as a



For peer review only - http://bmjopen.bmj.com/site/about/guidelines.xhtml

protective factor for the occurrence of CAA, with a pooled OR of 0.65 (95% CI, 0.54–0.77). For positive association, it was reported that hyperglycemia can trigger arterial inflammation and increase the risk of atherosclerosis,[36] which is a risk factor for CAA. Nonetheless, debate on whether CAA is a variant of atherosclerosis still exists.[38] Conversely, some authors proposed that the protective effect of diabetes may be from negative arterial remodeling[39] and increased matrix volume[37] of the coronary arteries. As a whole, the association between diabetes and CAA is currently not well-defined.

In our study, the results showed that there were positive association between aortic dissection, AA, and SLE and the presence of CAA. It had been speculated that CAA and AA share a similar histology and pathogenic process, which is generalized impairment of the wall of the entire arterial system.[40] The matrix metalloproteinases, a group of enzymes to degrade various components of the extracellular matrix in the arterial wall, have been found with elevated levels both in patients with CAA and those with AA.[41, 42] With respect to SLE, an increasing number of case reports have indicated its relationship with CAA in recent years.[43-46] In addition, SLE was found to be associated with AA.[47, 48] It was suggested that CAA resulted from severe coronary inflammation through the deposition of immune globulin and complement.[49,50] At other aspects, SLE was thought to induce accelerated atherosclerosis, which was a risk factor for aneurysms.[51] Although there were reports suggesting the relationship between SLE and development of CAA, the exact pathogenesis remains poorly



For peer review only - http://bmjopen.bmj.com/site/about/guidelines.xhtml

understood.

To the best of our knowledge, this was the first population-based study investigating the epidemiology and risk factors for CAA in Taiwan. Using a specific NHIRD dataset on all patients with CAA, which were able to provide an explicit age distribution, including both pediatric and adult populations. However, there were some limitations in the current study. First, we were unable to estimate the prevalence because not all patients in the epidemiologic study were available for follow-up. Second, our data were obtained from a claim-based database, which did not include information on aneurysm features, such as size, shape, location, number of aneurysms, degree of stenosis, and life style. In addition, we could not differentiate between aneurysm and ectasia in this study. Third, the control group selected from the general population may have had less opportunity to undergo examinations to detect atherosclerosis and AA. For this reason, the control group may have had fewer related events than the case group.

In conclusion, CAA patients were distributed both in pediatric and adult population in Taiwan. Kawasaki disease was predominant in children with CAA. Aside from the cardiovascular diseases, factors such as aortic disease and SLE, may predispose adults to develop CAA. The results of this study are limited by the lack of available data on process and effectiveness of management and medical therapy of CAA. Future studies are recommended to evaluate pharmacoepidemiology of CAA.



For peer review only

For peer review only - http://bmjopen.bmj.com/site/about/guidelines.xhtml

**Acknowledgements**

This study is based in part on data from the NHIRD provided by the Bureau of the NHI, Department of Health and managed by National Health Research Institutes. The authors acknowledge the assistance of the Bureau of the NHI and National Health Research Institutes. This work was supported by the Ministry of Science and Technology (MOST 104-2320-B-037-035) and Kaohsiung Medical University Hospital (KMUH105-M512).

**Author contributions**

Chein-Tang Fang, Yaw-Bin Huang, and Chung-Yu Chen designed the data collection instruments, conceptualized and designed the study, drafted the initial manuscript and approved the final manuscript as submitted. Chen-Chun Kuo, Chein-Tang Fang, Yi-Ping Fang and Chung-Yu Chen carried out the initial analyses, critically reviewed the manuscript, and all authors approved the final manuscript as submitted.

**Funding:** This work was supported by grants from Ministry of Science and Technology (MOST 104-2320-B-037-035) and Kaohsiung Medical University Hospital (KMUH105-M512)

**Competing interests:** None declared.

**Ethics approval:** This study was approved by the Institutional Review Board of Kaohsiung Medical University Hospital.

**Provenance and peer review:** Not commissioned; externally peer reviewed.

**Data sharing statement:** No additional data are available.

For peer review only

## References


1. Swaye PS, Fisher LD, Litwin P, Vignola PA, Judkins MP, Kemp HG, et al. Aneurysmal coronary artery disease. *Circulation*. 1983;67(1):134-8.
2. Tunick PA, Slater J, Kronzon I, Glassman E. Discrete atherosclerotic coronary artery aneurysms: a study of 20 patients. *J Am Coll Cardiol*. 1990;15(2):279-82.
3. Giannoglou GD, Antoniadis AP, Chatzizisis YS, Damvopoulou E, Parcharidis GE, Louridas GE. Prevalence of ectasia in human coronary arteries in patients in northern Greece referred for coronary angiography. *Am J Cardiol*. 2006;98(3):314-8.
4. Almansori MA, Elsayed HA. Coronary artery ectasia - A sample from Saudi Arabia. *J Saudi Heart Assoc*. 2015;27(3):160-3.
5. Wang KY, Ting CT, St John Sutton M, Chen YT. Coronary artery aneurysms: A 25-patient study. *Catheter Cardiovasc Interv*. 1999;48(1):31-8.
6. Yip HK, Chen MC, Wu CJ, Hang CL, Hsieh KY, Fang CY, et al. Clinical features and outcome of coronary artery aneurysm in patients with acute myocardial infarction undergoing a primary percutaneous coronary intervention. *Cardiology*. 2002;98(3):132-40.
7. Swanton RH, Thomas ML, Coltart DJ, Jenkins BS, Webb-Peploe MM, Williams BT. Coronary artery ectasia--a variant of occlusive coronary arteriosclerosis. *Br Heart J*. 1978;40(4):393-400.
8. Cohen P, O'Gara PT. Coronary artery aneurysms: a review of the natural history, pathophysiology, and management. *Cardiol Rev*. 2008;16(6):301-4.
9. Diaz-Zamudio M, Bacilio-Perez U, Herrera-Zarza MC, Meave-Gonzalez A, Alexanderson-Rosas E, Zambrana-Balta GF, et al. Coronary artery aneurysms and ectasia: role of coronary CT angiography. *Radiographics*. 2009;29(7):1939-54.
10. Uehara R, Belay ED. Epidemiology of Kawasaki disease in Asia, Europe, and the United States. *J Epidemiol*. 2012;22(2):79-85.
11. Lin MC, Lai MS, Jan SL, Fu YC. Epidemiologic features of Kawasaki disease in acute stages in Taiwan, 1997-2010: effect of different case definitions in claims data analysis. *J Chin Med Assoc*. 2015;78(2):121-6.
12. Manginas A, Cokkinos DV. Coronary artery ectasias: imaging, functional assessment and clinical implications. *Eur Heart J*. 2006;27(9):1026-31.
13. Antoniadis AP, Chatzizisis YS, Giannoglou GD. Pathogenetic mechanisms of coronary ectasia. *Int J Cardiol*. 2008;130(3):335-43.
14. Rath S, Har-Zahav Y, Battler A, Agranat O, Rotstein Z, Rabinowitz B, et al. Fate of nonobstructive aneurysmatic coronary artery disease: angiographic and clinical follow-up report. *Am Heart J*. 1985;109(4):785-91.
15. Kruger D, Stierle U, Herrmann G, Simon R, Sheikhzadeh A. Exercise-induced myocardial ischemia in isolated coronary artery ectasias and aneurysms ("dilated




For peer review only - http://bmjopen.bmj.com/site/about/guidelines.xhtml

coronopathy"). *J Am Coll Cardiol*. 1999;34(5):1461-70.
16. Sayin T, Doven O, Berkalp B, Akyurek O, Gulec S, Oral D. Exercise-induced myocardial ischemia in patients with coronary artery ectasia without obstructive coronary artery disease. *Int J Cardiol*. 2001;78(2):143-9.
17. Cheng CL, Chien HC, Lee CH, Lin SJ, Yang YH. Validity of in-hospital mortality data among patients with acute myocardial infarction or stroke in National Health Insurance Research Database in Taiwan. *Int J Cardiol*. 2015;201:96-101.
18. Nichols L, Lagana S, Parwani A. Coronary artery aneurysm: a review and hypothesis regarding etiology. *Arch Pathol Lab Med*. 2008;132(5):823-8.
19. Baman TS, Cole JH, Devireddy CM, Sperling LS. Risk factors and outcomes in patients with coronary artery aneurysms. *Am J Cardiol*. 2004;93(12):1549-51.
20. Saglam M, Karakaya O, Barutcu I, Esen AM, Turkmen M, Kargin R, et al. Identifying cardiovascular risk factors in a patient population with coronary artery ectasia. *Angiology*. 2007;58(6):698-703.
21. Amirzadegan AR, Davoodi G, Soleimani A, Lotfi Tokaldany M, Hakki Kazazi E, Shabpiray H, et al. Association between Traditional Risk Factors and Coronary Artery Ectasia: A Study on 10057 Angiographic Procedures among Iranian Population. *J Tehran Heart Cent*. 2014;9(1):27-32.
22. Androulakis AE, Andrikopoulos GK, Kartalis AN, Stougiannos PN, Katsaros AA, Syrogiannidis DN, et al. Relation of coronary artery ectasia to diabetes mellitus. *Am J Cardiol*. 2004;93(9):1165-7.
23. Androulakis AE, Katsaros AA, Kartalis AN, Stougiannos PN, Andrikopoulos GK, Triantafyllidi EI, et al. Varicose veins are common in patients with coronary artery ectasia. Just a coincidence or a systemic deficit of the vascular wall? *Eur J Vasc Endovasc Surg*. 2004;27(5):519-24.
24. Aqel RA, Zoghbi GJ, Iskandrian A. Spontaneous coronary artery dissection, aneurysms, and pseudoaneurysms: a review. *Echocardiography*. 2004;21(2):175-82.
25. Lamblin N, Gautier C, Le Tourneau T, Lablanche JM, Deklunder G, Bauters C. Prospective aortic screening in men with coronary aneurysms. *J Am Coll Cardiol*. 2006;47(6):1227-9.
26. Department of Household Registration, Ministry of the Interior. Midyear Population by Sex and 5-Year Age Group. [cited 2016 April 10]. Available from: http://www.ris.gov.tw/web/ris3-english/history.
27. Yang JJ, Yang X, Chen ZY, Wang Q, He B, Du LS, et al. Prevalence of coronary artery ectasia in older adults and the relationship with epicardial fat volume by cardiac computed tomography angiography. *J Geriatr Cardiol*. 2013;10(1):10-5.
28. Pinar Bermudez E, Lopez Palop R, Lozano Martinez-Luengas I, Cortes Sanchez R, Carrillo Saez P, Rodriguez Carreras R, et al. Coronary ectasia: prevalence, and clinical and



For peer review only - http://bmjopen.bmj.com/site/about/guidelines.xhtml

angiographic characteristics. *Rev Esp Cardiol*. 2003;56(5):473-9.

29. Sultana R, Sultana N, Ishaq M, Samad A. The prevalence and clinical profile of angiographic coronary ectasia. *J Pak Med Assoc*. 2011;61(4):372-5.

30. Boles U, Zhao Y, Rakhit R, Shiu MF, Papachristidis A, David S, et al. Patterns of coronary artery ectasia and short-term outcome in acute myocardial infarction. *Scand Cardiovasc J*. 2014;48(3):161-6.

31. Chang LY, Chang IS, Lu CY, Chiang BL, Lee CY, Chen PJ, et al. Epidemiologic features of Kawasaki disease in Taiwan, 1996-2002. *Pediatrics*. 2004;114(6):e678-82.

32. Huang WC, Huang LM, Chang IS, Chang LY, Chiang BL, Chen PJ, et al. Epidemiologic features of Kawasaki disease in Taiwan, 2003-2006. *Pediatrics*. 2009;123(3):e401-5.

33. Pahlavan PS, Niroomand F. Coronary artery aneurysm: a review. *Clin Cardiol*. 2006;29(10):439-43.

34. Gunes Y, Boztosun B, Yildiz A, Metin Esen A, Saglam M, Bulut M, et al. Clinical profile and outcome of coronary artery ectasia. *Heart*. 2006;92(8):1159-60.

35. Markis JE, Joffe CD, Cohn PF, Feen DJ, Herman MV, Gorlin R. Clinical significance of coronary arterial ectasia. *Am J Cardiol*. 1976;37(2):217-22.

36. Libby P. Inflammation in atherosclerosis. *Nature*. 2002;420(6917):868-74.

37. Huang QJ, Liu J, Chen MH, Li JJ. Relation of diabetes to coronary artery ectasia: A meta-analysis study. *Anadolu Kardiyol Derg*. 2014;14(4):322-7.

38. Yetkin E, Waltenberger J. Novel insights into an old controversy: is coronary artery ectasia a variant of coronary atherosclerosis? *Clin Res Cardiol*. 2007;96(6):331-9.

39. Dogan A, Arslan A, Yucel H, Aksoy F, Icli A, Ozaydin M, et al. Gamma glutamyltransferase, inflammation and cardiovascular risk factors in isolated coronary artery ectasia. *Rev Port Cardiol*. 2016;35(1):33-9.

40. Stajduhar KC, Laird JR, Rogan KM, Wortham DC. Coronary arterial ectasia: increased prevalence in patients with abdominal aortic aneurysm as compared to occlusive atherosclerotic peripheral vascular disease. *Am Heart J*. 1993;125(1):86-92.

41. Carrell TW, Burnand KG, Wells GM, Clements JM, Smith A. Stromelysin-1 (matrix metalloproteinase-3) and tissue inhibitor of metalloproteinase-3 are overexpressed in the wall of abdominal aortic aneurysms. *Circulation*. 2002;105(4):477-82.

42. Tengiz I, Ercan E, Aliyev E, Sekuri C, Duman C, Altuglu I. Elevated levels of matrix metalloprotein-3 in patients with coronary aneurysm: A case control study. *Curr Control Trials Cardiovasc Med*. 2004;5(1):10.

43. Yoshikai M, Hamada M, Takarabe K. Coronary artery aneurysm with systemic lupus erythematosus. *Jpn J Thorac Cardiovasc Surg*. 2004;52(8):379-82.

44. Suzuki H, Fujigaki Y, Mori M, Yamamoto T, Kato A, Wakahara N, et al. Giant coronary aneurysm in a patient with systemic lupus erythematosus. *Intern Med*. 2009;48(16):1407-12.

45. Posadzy-Malaczynska A, Woznicka-Leskiewicz L, Juszkat R, Frankiewicz M, Kociemba



For peer review only - http://bmjopen.bmj.com/site/about/guidelines.xhtml

A. Right coronary artery aneurysm with fistula into the coronary sinus in patient with systemic lupus erythematosus. *Kardiol Pol*. 2013;71(12):1329.

46. Monigari N, Poondru RR, Shetty RK, Vivek G. Giant coronary aneurysm presenting as acute coronary syndrome in a patient with systemic lupus erythematosus. *BMJ Case Rep*. 2014;2014.

47. Wang SH, Chang YS, Liu CJ, Lai CC, Chen TJ, Chen WS. Incidence and risk analysis of aortic aneurysm and aortic dissection among patients with systemic lupus erythematosus: a nationwide population-based study in Taiwan. *Lupus*. 2014;23(7):665-71.

48. Guy A, Tiosano S, Comaneshter D, Tekes-Manova D, Shovman O, Cohen AD, et al. Aortic aneurysm association with SLE - a case-control study. *Lupus*. 2016.

49. Miner JJ, Kim AH. Cardiac manifestations of systemic lupus erythematosus. *Rheum Dis Clin North Am*. 2014;40(1):51-60.

50. Famularo G, Vallone A, Compagnucci M, Capo G, Minisola G, Gasbarrone L. Tiny bubbles: coronary aneurysms and lupus. *Am J Med*. 2014;127(10):933-5.

51. Stojan G, Petri M. Atherosclerosis in systemic lupus erythematosus. *J Cardiovasc Pharmacol*. 2013;62(3):255-62.

For peer review only



For peer review only - http://bmjopen.bmj.com/site/about/guidelines.xhtml

## Figure Legends

Figure 1. Flow chart of the study population.

Figure 2. Age distribution of the CAA population

Figure 3. (a) Incidence rate of CAA population during 2005-2011. (b) Mortality rate of CAA population during 2005-2011

For peer review only



For peer review only - http://bmjopen.bmj.com/site/about/guidelines.xhtml

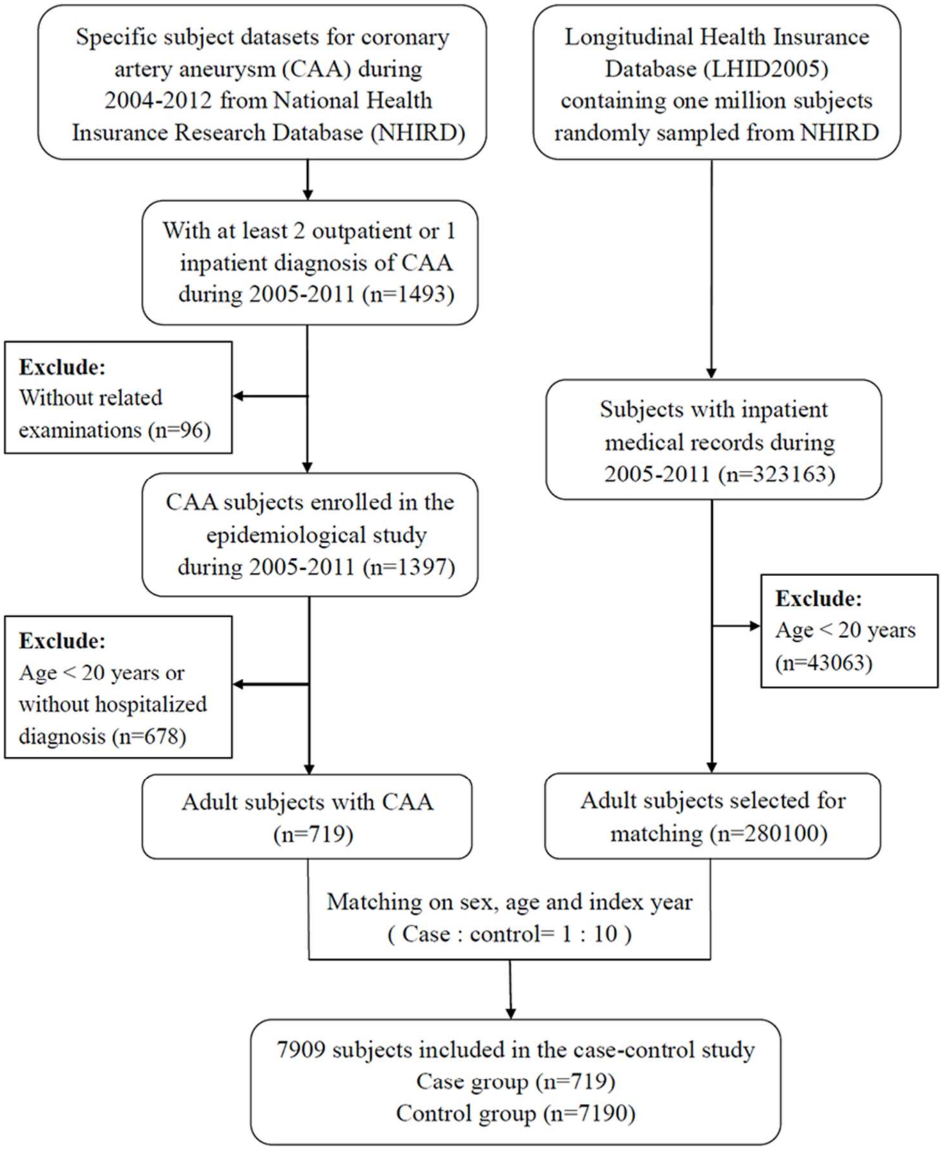


78x94mm (300 x 300 DPI)

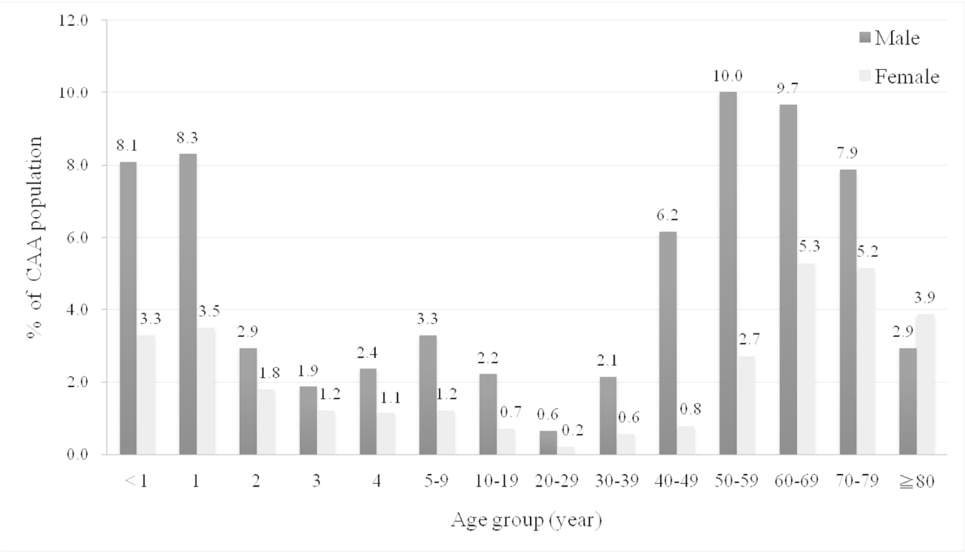


194x112mm (300 x 300 DPI)

For peer review only

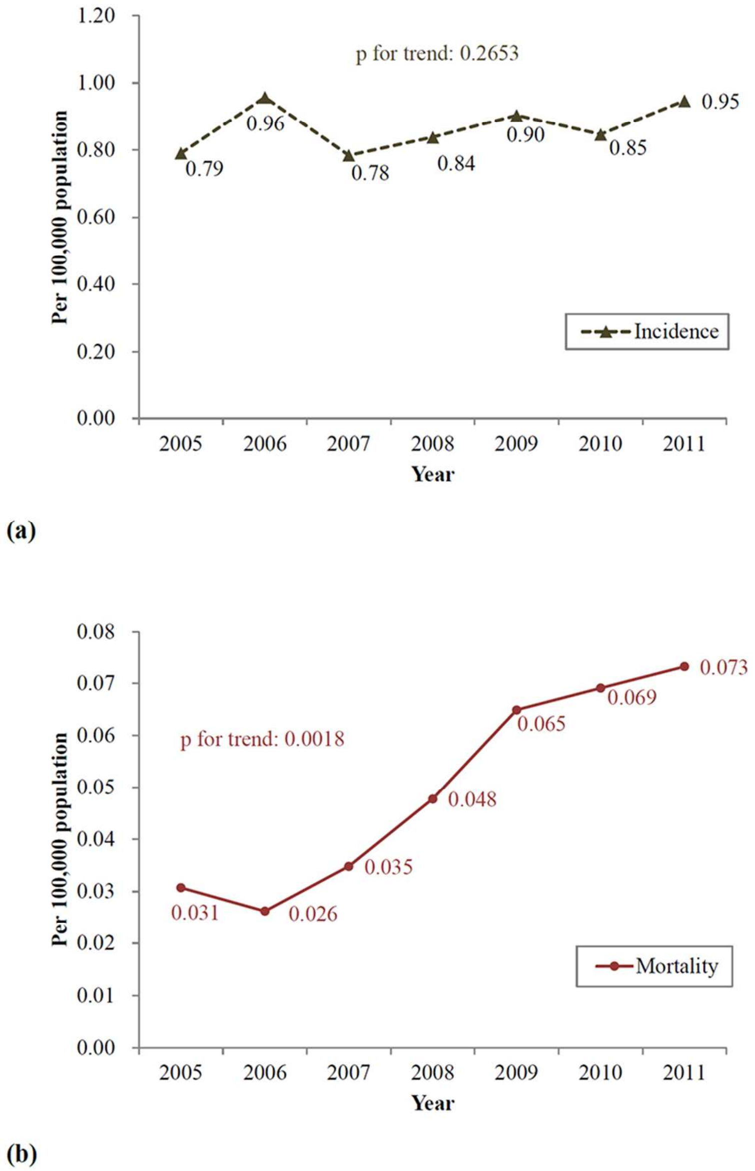


69x106mm (300 x 300 DPI)

# Supplementary

eTable 1. Spearman correlation coefficients among risk factors

| | | DM | CVD | PVD | VV | AD | AA | SLE | RA | IBD |
|---|---|---|---|---|---|---|---|---|---|---|
| **CAS** | r | 0.103* | 0.036* | 0.023* | 0.002 | 0.030* | 0.021 | 0.010 | 0.015 | -0.011 |
| | p | **<0.0001** | **<0.05** | **<0.05** | 0.873 | **<0.05** | 0.068 | 0.397 | 0.176 | 0.342 |
| **HTN** | r | 0.284* | 0.198* | 0.028* | -0.008 | 0.035* | 0.040* | 0.016 | 0.019 | 0.007 |
| | p | **<0.0001** | **<0.0001** | **<0.05** | 0.483 | **<0.05** | **<0.001** | 0.149 | 0.092 | 0.549 |
| **Lipid** | r | 0.267* | 0.015 | 0.036* | -0.012 | -0.007 | 0.010 | 0.002 | 0.007 | -0.025* |
| | p | **<0.0001** | 0.178 | **<0.05** | 0.301 | 0.515 | 0.375 | 0.831 | 0.532 | **<0.05** |

r, Spearman correlation coefficient; * $p < 0.05$;

CAS, coronary atherosclerosis; HTN, hypertension; Lipid, Dyslipidemia; DM, diabetes mellitus; CVD, cerebrovascular disease; PVD, peripheral vascular disease; VV, varicose vein; AD, aortic dissection; AA, aortic aneurysm; SLE, systemic lupus erythematosus; RA, rheumatoid arthritis; IBD, inflammatory bowel disease.

**STROBE 2007 (v4) Statement—Checklist of items that should be included in reports of *cohort studies***

| Section/Topic | Item # | Recommendation | Reported on page # |
|---|---|---|---|
| **Title and abstract** | 1 | (*a*) Indicate the study's design with a commonly used term in the title or the abstract | 2 |
| | | (*b*) Provide in the abstract an informative and balanced summary of what was done and what was found | 2 |
| **Introduction** | | | |
| Background/rationale | 2 | Explain the scientific background and rationale for the investigation being reported | 4 |
| Objectives | 3 | State specific objectives, including any prespecified hypotheses | 4 |
| **Methods** | | | |
| Study design | 4 | Present key elements of study design early in the paper | 5-7 |
| Setting | 5 | Describe the setting, locations, and relevant dates, including periods of recruitment, exposure, follow-up, and data collection | 5-7 |
| Participants | 6 | (*a*) Give the eligibility criteria, and the sources and methods of selection of participants. Describe methods of follow-up | 5-6 |
| | | (*b*) For matched studies, give matching criteria and number of exposed and unexposed | 5-6 |
| Variables | 7 | Clearly define all outcomes, exposures, predictors, potential confounders, and effect modifiers. Give diagnostic criteria, if applicable | 5-7 |
| Data sources/ measurement | 8* | For each variable of interest, give sources of data and details of methods of assessment (measurement). Describe comparability of assessment methods if there is more than one group | 5 |
| Bias | 9 | Describe any efforts to address potential sources of bias | 5-6 |
| Study size | 10 | Explain how the study size was arrived at | 5-6 |
| Quantitative variables | 11 | Explain how quantitative variables were handled in the analyses. If applicable, describe which groupings were chosen and why | 5 |
| Statistical methods | 12 | (*a*) Describe all statistical methods, including those used to control for confounding | 8 |
| | | (*b*) Describe any methods used to examine subgroups and interactions | 8 |
| | | (*c*) Explain how missing data were addressed | 8 |
| | | (*d*) If applicable, explain how loss to follow-up was addressed | 8 |
| | | (*e*) Describe any sensitivity analyses | 8 |
| **Results** | | | |

For peer review only

| | | | |
|---|---|---|---|
| Participants | 13* | (a) Report numbers of individuals at each stage of study—eg numbers potentially eligible, examined for eligibility, confirmed eligible, included in the study, completing follow-up, and analysed | 8-14 |
| | | (b) Give reasons for non-participation at each stage | 8-14 |
| | | (c) Consider use of a flow diagram | 9 |
| Descriptive data | 14* | (a) Give characteristics of study participants (eg demographic, clinical, social) and information on exposures and potential confounders | 10 |
| | | (b) Indicate number of participants with missing data for each variable of interest | 11 |
| | | (c) Summarise follow-up time (eg, average and total amount) | 10 |
| Outcome data | 15* | Report numbers of outcome events or summary measures over time | 8-14 |
| Main results | 16 | (*a*) Give unadjusted estimates and, if applicable, confounder-adjusted estimates and their precision (eg, 95% confidence interval). Make clear which confounders were adjusted for and why they were included | 8-14 |
| | | (*b*) Report category boundaries when continuous variables were categorized | 8-14 |
| | | (*c*) If relevant, consider translating estimates of relative risk into absolute risk for a meaningful time period | 8-14 |
| Other analyses | 17 | Report other analyses done—eg analyses of subgroups and interactions, and sensitivity analyses | 8-14 |
| **Discussion** | | | |
| Key results | 18 | Summarise key results with reference to study objectives | 15 |
| **Limitations** | | | 19 |
| Interpretation | 20 | Give a cautious overall interpretation of results considering objectives, limitations, multiplicity of analyses, results from similar studies, and other relevant evidence | 19 |
| Generalisability | 21 | Discuss the generalisability (external validity) of the study results | 16-19 |
| **Other information** | | | |
| Funding | 22 | Give the source of funding and the role of the funders for the present study and, if applicable, for the original study on which the present article is based | 20 |

*Give information separately for cases and controls in case-control studies and, if applicable, for exposed and unexposed groups in cohort and cross-sectional studies.

**Note:** An Explanation and Elaboration article discusses each checklist item and gives methodological background and published examples of transparent reporting. The STROBE checklist is best used in conjunction with this article (freely available on the Web sites of PLoS Medicine at http://www.plosmedicine.org/, Annals of Internal Medicine at http://www.annals.org/, and Epidemiology at http://www.epidem.com/). Information on the STROBE Initiative is available at www.strobe-statement.org.

For peer review only
